# MicroRNA Genes Derived from Repetitive Elements and Expanded by Segmental Duplication Events in Mammalian Genomes

**DOI:** 10.1371/journal.pone.0017666

**Published:** 2011-03-16

**Authors:** Zhidong Yuan, Xiao Sun, Hongde Liu, Jianming Xie

**Affiliations:** 1 State Key Laboratory of Bioelectronics, School of Biological Science and Medical Engineering, Southeast University, Nanjing, China; 2 School of Life Science, Hunan University of Science and Technology, Xiangtan, China; University Hospital Frankfurt, Germany

## Abstract

MicroRNAs (miRNAs) are a class of small noncoding RNAs that regulate gene expression by targeting mRNAs for translation repression or mRNA degradation. Many miRNAs are being discovered and studied, but in most cases their origin, evolution and function remain unclear. Here, we characterized miRNAs derived from repetitive elements and miRNA families expanded by segmental duplication events in the human, rhesus and mouse genomes. We applied a comparative genomics approach combined with identifying miRNA paralogs in segmental duplication pair data in a genome-wide study to identify new homologs of human miRNAs in the rhesus and mouse genomes. Interestingly, using segmental duplication pair data, we provided credible computational evidence that two miRNA genes are located in the pseudoautosomal region of the human Y chromosome. We characterized all the miRNAs whether they were derived from repetitive elements or not and identified significant differences between the repeat-related miRNAs (RrmiRs) and non-repeat-derived miRNAs in (1) their location in protein-coding and intergenic regions in genomes, (2) the minimum free energy of their hairpin structures, and (3) their conservation in vertebrate genomes. We found some lineage-specific RrmiR families and three lineage-specific expansion families, and provided evidence indicating that some RrmiR families formed and expanded during evolutionary segmental duplication events. We also provided computational and experimental evidence for the functions of the conservative RrmiR families in the three species. Together, our results indicate that repetitive elements contribute to the origin of miRNAs, and large segmental duplication events could prompt the expansion of some miRNA families, including RrmiR families. Our study is a valuable contribution to the knowledge of evolution and function of non-coding region in genome.

## Introduction

Several hypotheses for the origin and formation of new microRNA (miRNA) genes have been proposed [1]–[3]. MiRNA genes may originate from inverted duplication (mainly found in plants) [4]–[7], tandem duplications (also called local duplication) [8]–[10], segmental duplications (SDs) [9],[11], random hairpin structures located in intronic or intergenic regions [12], or from repetitive elements especially transposable elements (TEs) [13]–[20]. Repetitive elements are multiple copies of DNA sequences present in the same genome and also classified as tandem arrays or interspersed repeats [21], [22]. Tandem arrays include satellites, telomeric repeats, subtelomeric repeats, microsatellites and minisatellites. Interspersed repeats encompass TEs (DNA transposon, LTR retrotransposons and non-LTR retrotransposons) and processed pseudogenes [21], [23]. Some SDs (intrachromosomal duplications) are also referred to as low copy repeats [24], [25]. Repeat sequences are prevalent in the genomes of all plants and animals. For example, repeat sequences make up more than 50% of the human genome [24], and TEs account for 45%, 40%, 15–22%, 12%, and 8.6% of the human, mouse, fruit fly, nematode and chicken genomes, respectively [24], [26]–[28]. In some plants, TEs constitute up to 90% of the genome [27].

TEs are attracting more attention than before because more and more evidence shows that TEs play a major role in shaping the structure and function of the genome. TEs, when inserted and integrated upstream of a gene, may change the expression pattern of the gene [29]; in an exon they may produce a new protein domain [29], [30]; and when inserted into an intron TE may to produce a *de novo* protein [31], [32]. There is also some evidence to suggest that repeats may have contributed to the birth of miRNAs. That some miRNAs are derived from genome repeats in both sense and antisense directions was first documented in *Arabidopsis thaliana* [33]. In animals, repeat-derived miRNAs (RdmiRs) were first discovered in the human, mouse and rat genomes. Initially, only 7, 9 and 10 miRNA genes containing repeat sequences were found in the human, mouse and rat genomes, respectively [19]. Later, Piriyapongsa et al reported 68 human miRNA genes that share sequences with TEs, and discovered 55 TE-derived miRNAs among 462 human miRNA genes (miRBase 8.2) [18]. Now a lot more evidence is available to support the hypothesis that miRNAs could be derived from TEs in plants and animals [13]–[20].

Copy-number variant (CNV) is defined as a DNA segment at least 1 kb in length, in which copy number differences have been observed by comparison of two or more genomes [34]. Some CNVs, when fixed in a population, give rise to partial SDs [35], [36]. SDs are segments of DNA >1 kb in length that occur in two or more copies per haploid genome, with the different copies sharing >90% sequence identity [37]. Although previous reports have mentioned that novel miRNAs can be produced from miRNA gene duplication [4]–[10], there is little known about the miRNAs that are produced and expanded in SD events. We hypothesized that, like protein-coding genes, miRNAs (including RrmiRs) may also duplicate and expand accompanying large genomic SD events in their evolutionary history. We tested this hypothesis and found that SD pair data are helpful in identifying some miRNA paralogs. We have defined a target SD as the SD that is duplicated from another SD, the source SD. A source and its target are defined as a SD pair.

In this study, we present a systematic study for the miRNAs derived from repetitive elements and expanded in the SD events in human, rhesus and mouse.

## Materials and Methods

### A genome-scale combinational method for detecting homologous miRNA

The miRNAs used in this study were downloaded from miRBase 16 (Sept 2010) [38]. The current miRBase contains 1,048 miRNA genes and 1,223 mature sequences from human, 672 miRNA genes and 1,055 mature sequences from mouse, and 466 miRNA genes and 488 mature sequences from rhesus. As in miRBase [40] and in previous reports [16]–[18], [39], the hairpin sequences of miRNAs are referred to as miRNA genes [16]–[18], [39] and their loci as microRNA gene loci [40]. SD data are all from the UCSC Genome Browser [41]. To utilize the SD pair data from the mm8 mouse genome assembly, we translated the coordinates of the miRNA genes from the mm9 mouse assembly to the mm8 assembly using the liftOver utility [42] provided by UCSC Genome Browser [41]. Because of a gap in the corresponding genomic region in the mm8 assembly, 7 miRNAs from the mir-290 family could not be mapped. These miRNAs are not included in the following analysis. Some in-house scripts were also used to process data.

We developed a combinatorial method to identify homologous miRNA genes. First, we obtained the whole genome pairwise alignment (human vs rhesus and human vs mouse) data from the UCSC Genome Browser [41]. These alignment data produced by the LASTZ (a replacement for BLASTZ [43]), chain and net program (http://hgdownload.cse.ucsc.edu/admin/jksrc.zip). We then used the alignment data to map the human miRNA genes to the rhesus and mouse genomes with the LiftOver utility [42] to obtain the genome coordinates of potential miRNA genes in the two genomes. Next, we filtered out the potential miRNA genes that were shorter than 39 bps or longer than 215 bps (These two values were determined based on the current miRBase sequence data for animals). The sequences of the preliminary potential miRNA genes were retrieved and classified using the MiPred program [44]. The region where several genome coordinates of miRNA genes overlapped (mapped from the human miRNA coordinates usually from the same miRNA family), was considered to be the only valid miRNA genome coordinate and is the only one used in the present study. Finally, potential miRNA paralogs in SD pairs were identified and classified with MiPred [44]. The newly identified orthologous and paralogous sequences of human miRNA genes were named according to the miRBase naming criteria [38].

### Genome-wide analysis of repeat-derived miRNAs

Repeats were annotated with the RepeatMasker program [45] and the genomic positions of repeats were taken from UCSC Genome Browser [41] using the Table Browser [46]. The coordinates of mature miRNAs were calculated according to their host miRNA genes. We used these coordinates to identify all miRNA genes overlapping with repeat sequences with Galaxy [47] and in-house scripts. The data were analyzed using a suite of functions written in R (version 2.9.0) [48]. If the coverage density of repetitive elements was at least 50% in a miRNA gene or 100% in one of the associated mature miRNA sequences, then the miRNA gene was considered to be a RdmiR [18]. To conveniently analyze the data, the miRNAs with a low coverage density of repeats (coverage density of repetitive elements >0% and <50% in the miRNA gene and <100% in its mature miRNA) were called possible repeat-derived miRNAs (PRdmiRs). All RdmiRs and PRdmiRs were determined by this criterion, regardless of whether the miRNA genes were locate on the same strand or on the opposite strand to the overlapping repetitive element. In this study, the RdmiRs and PRdmiRs are both referred to as repeat-related miRNAs (RrmiRs). The other miRNAs with no overlapping repeats are the NRdmiRs.

All the coordinates of the annotated genes of the three organisms were downloaded from the knownGene and refGene tables in the UCSC Genome Browser [41], non-protein coding gene information was removed, and the difference in distribution of RrmiRs and NRdmiRs was determined. As in previous reports [49], [50], all the miRNAs were classified into intragenic and intergenic miRNAs. The minimum free energy (MFE) of the hairpin structures for the three pre-miRNA types (RrmiRs, PRrmiRs and NRdmiRs) from the three species was calculated using the RNAfold program [51]. If the sequences from the three species were identical, only one from each miRNA family was retained.

### Calculation of conservation scores and miRNAs analysis in duplication data

Per-site conservation scores between 0 and 1 were calculated by phastCons program [52] based on the 17-species multiz alignment data [53] downloaded from the UCSC Genome Browser [41]. The 17-species include human, chimp, rhesus, mouse, rat, rabbit, dog, cow, armadillo, elephant, tenrec, opossum, chicken, frog, zebrafish, tetraodon and fugu. Because the only available conservation data for human are at Galaxy [47], and because they also include information for rhesus and mouse, we used the human miRNA genes to calculate the conservation scores for the NRdmiRs, PRdmiRs and RdmiRs. The total score and the average score of each pre-miRNA were calculated according to the single-nucleotide conservation score. To determine the lineage-specific miRNA families, the conservation scores of miRNAs, the paralogs and orthologs of miRNAs from miRBase, the new miRNAs discovered in rhesus, mouse and the other species were all taken into consideration.

To investigate if RrmiRs expansion accompanied SD events, we compared the genomic coordinates of the RrmiRs and SDs. CNV data have also been used to analyze the evolution of miRNAs. Human CNV data, which contain 1,445 copy number polymorphisms (CNPs), is from Redon et al. [36]. The UCSC liftOver program was used to convert human hg18 assembly coordinates for the CNV data to the hg19 assembly coordinates. The mouse and rhesus CNV data used in the analysis were obtained from the published data [54], [55].

### Target prediction, functional enrichment analysis and functional network construction

The 3′UTR sequences of reference protein-coding genes of human, rhesus and mouse were downloaded from the UCSC Genome Browser. If there were multiple variants of the 3′UTR sequences, then the longest one for each of the protein coding genes was retained. Common targets of the conservative RrmiR families were predicted using the PITA [56] and miRanda programs [57]. Enriched Gene Ontology (GO) terms for the targets were analyzed with the Bioconductor package topGO [58]. To identify which of the functions were validated by biological experiments, we searched the literature in Pubmed (up to 8 December 2010) for the miRNA entities. We integrated this information with the data from miRTarBase release 1.0 that curates experimentally validated microRNA-target interactions [59], and constructed functional interaction networks using Cytoscape v2.7.0 [60].

## Results

### Novel orthologs and paralogs of human miRNAs identified in the rhesus and mouse genome

The rhesus and human genomes diverged ∼25 million years ago and have 93% identity [61]. The mouse and human genomes diverged ∼75 million years ago and more than 90% of their genomes can be divided into corresponding regions of conserved synteny [62]. Because the divergence rate is low, the orthologous sequences of many functionally important elements (including the miRNA genes) can be aligned and identified and their cross-species conservation can be investigated [62].

**Figure 1:**
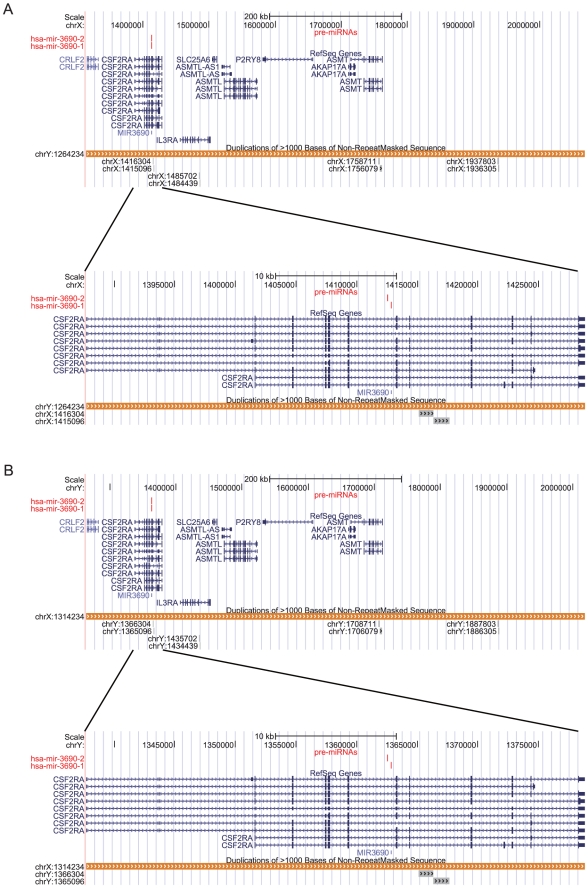
A representation of the location of the human miR-3690 gene in the duplicated pseudoautosomal regions of chromosomes X and Y. (A) chrX: 1314235–2068238 (+). (B) chrY: 1264235–2018238 (+).

We have identified 228 novel miRNA homologs in the rhesus genome (Mmul1.0 assembly) and 22 novel miRNA homologs in the mouse genome (mm9 assembly) (Text S1) using the method described in the Materials and Methods section. Based on the SD pair data, we found 12 novel miRNA paralogs in the human genome and two miRNA paralogs in the mouse genome; however, none were found in the rhesus genome (Text S1). There is an SD pair (chrX: 1314235–2068238 (+) and chrY: 1264235–2018238 (+)) in the corresponding pseudoautosomal region of the two sex chromosomes and the DNA sequences of the two segments are identical. Interestingly, the known miR-3690 gene [63] is located in the SD region on chrX: (1314235–2068238 (+)). No miRNA genes have yet been found on Y chromosome [40], but, using the SD pair data, we identified hsa-mir-3690-1and hsa-mir-3690-2 on both the X and Y chromosomes (Hsa-mir-3690 is documented in miRBase 16 and one new paralog of it has been found. We have renamed them according to the miRNA name criteria (Text S1)). Two duplicate copies of hsa-mir-3690 are located in an intron of the CSF2RA gene in the pseudoautosomal region of the X and Y chromosomes (Figure 1). In the same intron of CSF2RA gene (Figure 1), we found 15 duplicate copies of hsa-mir-3690 which was first documented in miRBase 16; only 2 of the copies were identified by MiPred [44] as potential miRNA genes, 7 were classified as pseudo miRNA genes and others were not miRNA genes (data not show). The composition and order of the genes in the the pseudoautosomal regions that contain the SD pair are shown in Figure 1. From this we can infer that duplicate copies of hsa-mir-3690 arose before crossover (which creates SD pairs) occurred in the pseudoautosomal regions of the X and Y chromosomes.

### Repeat related miRNAs

We have found 278 miRNA genes (226 RdmiRs and 52 PRdmiRs) in the human genome that overlap with repeats (Table S1). A recent paper that focused on TE-derived miRNAs, reported 68 human miRNA genes that shared sequences with TEs and 55 miRNA genes that were TE-derived [18]. Most of the TE-derived miRNAs that were reported in the earlier work were also identified as RdmiRs in the present study. Exceptions to this were hsa-mir-130b and hsa-mir-648 that we classified as NRdmiRs, and hsa-mir-659 that was classified as a PRdmiR. In our study, we used the data processed by the updated RepeatMasker program and the data from the updated miRBase. This may explain the discrepancies between our results and the results from the earlier study. In addition to miRNAs that overlap with TEs, we found many miRNA genes that overlap with other types of repetitive elements (Table S1). We also identified 141 RrmiRs (115 RdmiRs and 26 PRdmiRs) in the rhesus genome and 168 RrmiRs (141 RdmiRs and 27 PRdmiRs) in the mouse genome (Table S1).

**Figure 2:**
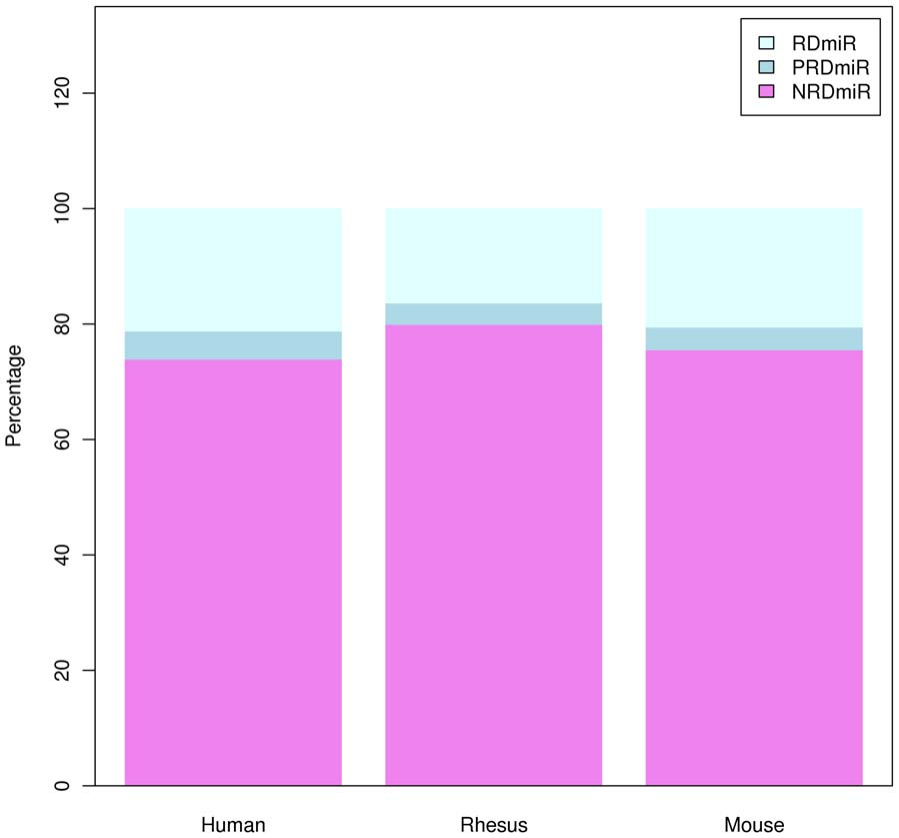
The percentages of NRdmiRs, PRdmiRs and RdmiRs in the human, rhesus and mouse genomes.

**Figure 3:**
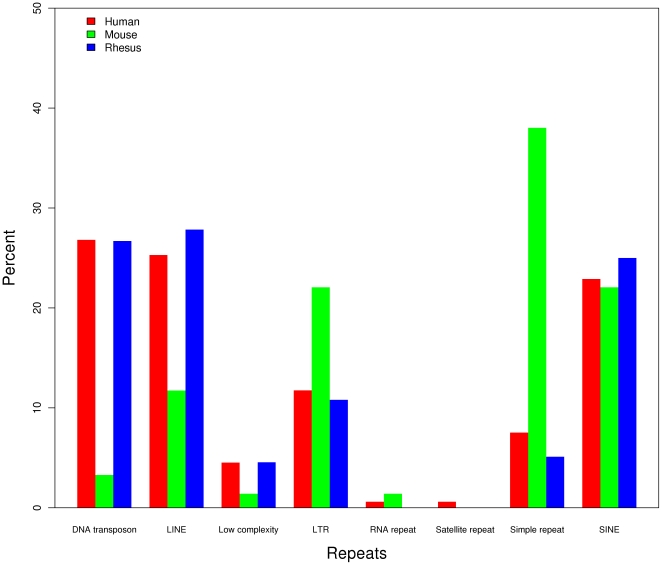
The distribution of repetitive elements types in repeat-related miRNAs in different species.

RrmiRs not only differ in terms of the coverage density of repeat sequences, but also in the number of different repeats from which they are derived (Figure 2 and Table S1). In the two primate species, most of the RrmiRs are derived from TEs that include DNA repeats, LINE, SINE and LTR while in the mouse genome, and most RrmiRs are from simple repeats followed by LTR, SINE, and LINE (Figure 3). The most abundant repetitive element types from which the human and rhesus miRNAs are derived are the DNA transposons (Figure 3), and the most abundant of those are the MADE1 elements belonging to the TcMar-Mariner family (Table S1). It is remarkable that the 42 human miRNA genes and the 25 rhesus miRNA genes that share sequences with MADE1 elements are all members of the miRNA-548 family (Table S1). In mouse, the most abundant repetitive element type from which the miRNAs are derived is the simple repeats ((CA)n and (TG)n). These simple repeats have produced the largest miRNA family (mir-467 family) in mouse during the evolutionary process.

### Differences between NRdmiRs, PRdmiRs and RdmiRs

**Figure 4:**
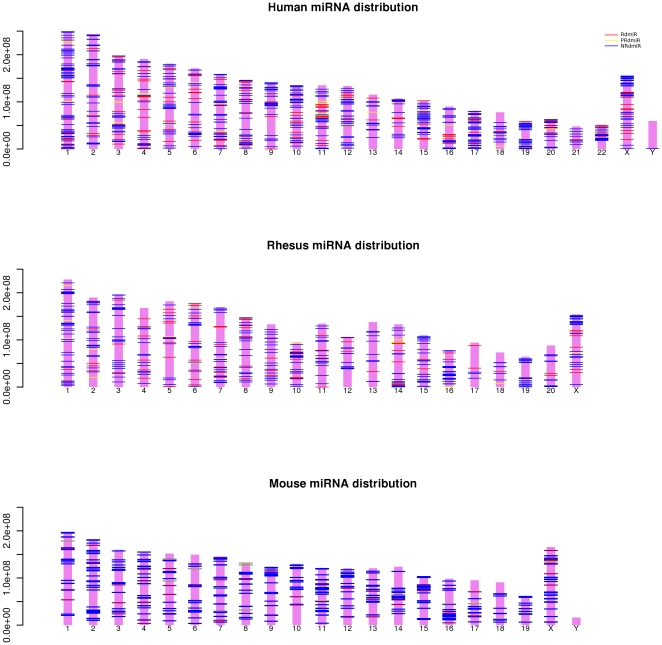
The distribution of the NRdmiRs, PRdmiRs and RdmiRs in the human, rhesus and mouse genome.

The genomic distribution of NRdmiRs, PRdmiRs and RdmiRs in human, rhesus and mouse is shown in Figure 4. There are no length data available for the Y chromosome of rhesus, making it impossible to draw it and the miRNAs mapping to the genome scaffolds or to the unplaced contigs also have not been plotted. As described earlier [64]–[68], the miRNA genes in animals tend to occur in clusters (Figure 4). We classified miRNAs into intragenic miRNAs and intergenic miRNAs and found that NRdmiRs and RrmiRs show significant different distributions in protein-coding genes compared to intergenic regions (human: chi-square test p-value  = 0.004131; rhesus: chi-square test p-value = 0.03475; mouse: chi-square test p-value = 2.1e-07).

**Figure 5:**
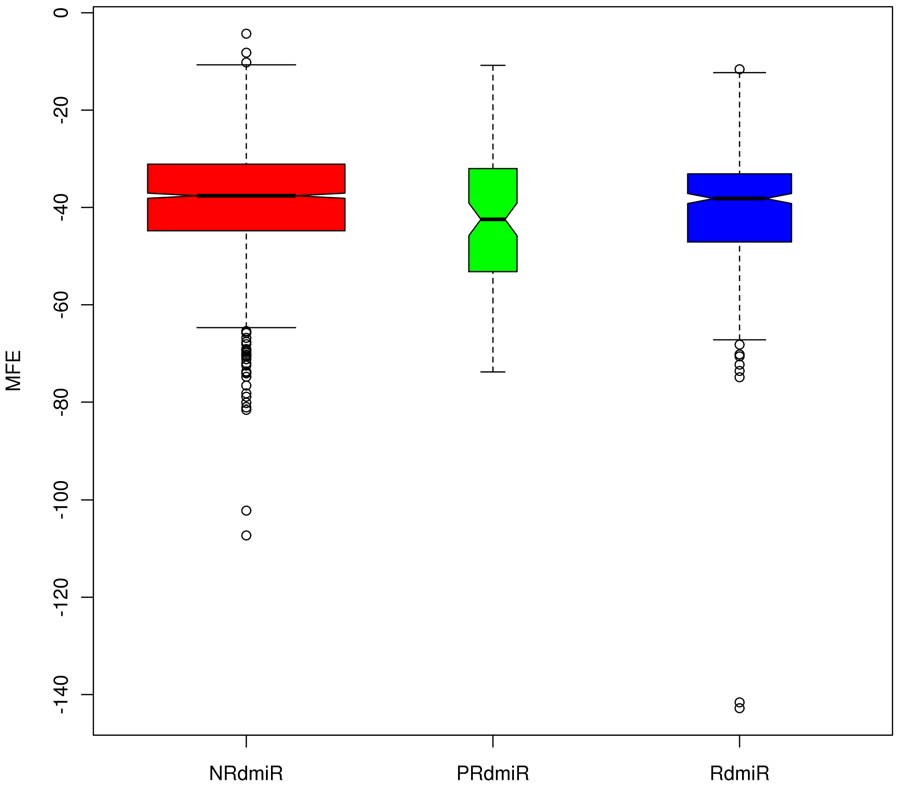
The MFEs for the miRNA precursors in the NRdmiRs, PRdmiRs and RdmiRs. The open circles indicate outliers.

To further explore the differences between the RdmiRs, PRdmiRs and NRdmiRs, we calculated their MFE structures using the RNAfold program [51] to determine the MFEs of hairpin structures for the three pre-miRNAs types in the three species. We found that the NRdmiRs, PRdmiRs and RdmiRs have significantly different MFE values (Figure 5, Kruskal-Wallis chi-squared  = 13.2434, df = 2, p-value  = 0.001331). The MFE values for NRdmiRs are significantly different from those for PRdmiRs (W = 97326.5, p-value  = 0.007105, Wilcoxon rank sum test) and RdmiRs (W = 422230.5, p-value = 0.006562, Wilcoxon rank sum test). No significant difference in MFE values was found between PRdmiRs and RdmiRs (W = 21293.5, p-value  = 0.1866, Wilcoxon rank sum test).

**Figure 6:**
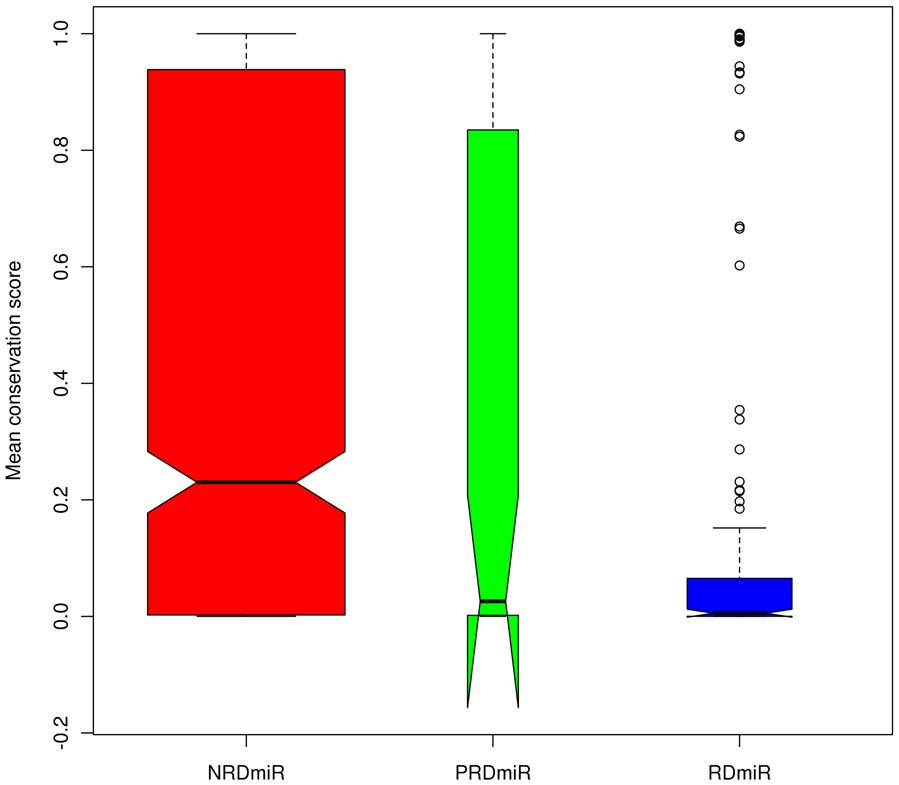
The average per-site conservation score for the NRdmiRs, PRdmiRs and RdmiRs. The open circles indicate outliers.

We evaluated the sequence conservation of the miRNAs based on the whole genome alignments of human, rhesus, mouse and 14 other vertebrate species by calculating the per-site conservation probability of the human pre-miRNAs. However, hsa-mir-1268 (derived from AluJo), hsa-mir-1299 (derived from CER), hsa-mir-3673 (derived from (TA)n), hsa-mir-3669 (derived from (CATATA)n) and hsa-mir-3683 (derived from SATR1) were not included in the calculation, because the orthologs of these miRNAs are not found in any of the other aligned animal genomes. Hsa-mir-1268 and hsa-mir-1299 do have homologs in chimpanzee (ptr-mir-1299) and orangutan (ppy-mir-1268 and ppy-mir-1299) that are documented in miRBase 16. This indicates that these two miRNAs are primate-specific miRNAs, and suggests that hsa-mir-3673, hsa-mir-3669 and hsa-mir-3683 may be human-specific miRNAs. The average conservation scores of NRdmiRs, PRdmiRs and RdmiRs are 0.4320636, 0.3406138 and 0.1585666 respectively, Figure 6 indicating significant differences in their conservation (Kruskal-Wallis chi-squared = 64.5498, df = 2, p-value = 9.62e-15). There is no significant difference between the conservation scores of NRdmiRs and PRdmiRs (W = 22320, p-value  = 0.2510, Wilcoxon rank sum test). The significant differences in conservation scores are between PRdmiRs and RdmiRs (W = 7364.5, p-value  = 0.001523, Wilcoxon rank sum test) and between NRdmiRs and RdmiRs (W = 117123.5, p-value  = 1.110e-15, Wilcoxon rank sum test) (Figure 6).

### Lineage-specific characters of the RrmiR families

Recent studies indicate that repetitive elements (especially TEs) have driven genome evolution in diverse ways, some of which tend to be lineage-specific [69]–[72]. For example, the mir-548 family (derived from MADE1 element) is primate-specific [16] and the mir-1302 family (derived from MER53 element) is a placental-specific miRNA family [20]. Similarly, we have found quite a few RrmiR families that are lineage-specific. Some larg families, the mir-506, mir-1972, mir-3118 and mir-3179 families are primate-specific. Although, in the present study, the mir-1285, mir-1289 and mir-3116 families are only appear in human, the mir-1285 and mir-3116 families are actually primate-specific miRNA families, and the mir-1289 family is not limited to primate species, but is also found in horse (eca-mir-1289). We also identified one murine-specific families, mir-1906 family, and some large families, for instance, mir-1195, mir-1937, mir-3470 and mir-3471 families are mouse-specific (Table S2). Three miRNA families that are the results of lineage-specific expansion were found in the mouse genome: the mir-466 and mir-467 families derived from simple repeats and the mir-297 family derived from SINE and LTR repetitive elements (Table S1 and Table S2). Although the mir-1255 family is a large miRNA family that includes members from primate and horse, no homologs have been found in mouse. In addition, there are quite a few small lineage-specific families, such as mir-1268, mir-1299, mir-3673 (human), mir-3669 (human), and mir-3683 (human) that may be expanded as more data become available (Table S2).

### MiRNAs in segmental duplication and the expansion of some miRNA families promoted by segmental duplication events

It is well known that gene duplication can involve inverted, local (tandem duplication) or segmental duplication events. That many miRNA genes originated from inverted duplication, and that miRNA families expanded through local duplication (tandem duplication), has been well characterized [4]–[10]. However, little is known about the expansion of miRNA genes and miRNA families through segmental duplications.

SD pairs are found as arrays of local duplications or dispersed between or within chromosomes. In the human genome, 55 NRdmiR genes, 4 PRdmiR genes and 22 RdmiR genes are located completely within SDs (Table S3). These 81 miRNA genes (distributed at 90 loci in the genome) include 69 miRNA genes in 59 SD pairs, and 12 miRNA genes for which the homologs have not been found in the corresponding SD pairs (Table S3 and Table S4). We also found that 20 of the miRNA genes (distributed at 22 loci in the genome) overlap with SDs in the rhesus genome. Except for mml-mir-372, which is not completely located within its corresponding SD, all the other rhesus miRNA genes are located in SDs (Table S3). The 20 rhesus miRNA genes include 16 miRNA genes in SD pairs, while the homologs of the other 4 miRNA genes have not been discovered in the corresponding SD pairs (Table S3 and Table S4). In the mouse genome, we only found 53 miRNA genes completely located in SDs and two (mmu-mir-367, mmu-mir-669g) partially overlap with SDs. Among the 55 mouse miRNA genes (Table S3), there are 46 miRNA genes in SD pairs and 9 miRNA genes that are found in only one SD of the corresponding SD pair and no homolog in the other SD of the pair (Table S3 and Table S4). It is interesting that most mouse miRNA genes in SDs and in SD pairs are derived from simple repeats in chromosome 2 (Table S1, Table S3 and Table S4).

When a miRNA gene is in a SD pair, we have identified three situations that may occur: (1) the miRNA gene has at least one paralog, (2) the miRNA gene has a homolog that is not a miRNA, and (3) the corresponding homolog segment is absent in the other SD of the same SD pair. Based on the SD pair data, we identified 12 potential miRNA genes in human and two potential miRNA genes in mouse (Text S1). The paralogs of only 25 miRNA genes in SD blocks from the three species were not detected in SD pairs. Two possible explanations and evolutionary scenarios might explain these results: (1) these regions originally harbored miRNAs that duplicated from one of the SDs of the SD pair and one of the genes has since degenerated, or (2) the miRNAs in the SD regions were gained after the SD duplication event.

When a miRNA family has only one miRNA gene in an SD pair, this implies that no homologs of the gene have been identified in the other SD of the SD pair. Then the expansion of this miRNA family could not have been promoted by early SD events in the evolutionary process. But if the members from a miRNA family are present in the two SDs of an SD pair, it is likely that the miRNA family was expanded by the early SD events. We found NRdmiR and RrmiR families that may have expanded by SD events. There are 16 known miRNA families (and 17 miRNAs that have not yet been classified to a known miRNA family in miRBase or Rfam) in the human genome, 5 known miRNA families in the mouse genome and 6 known miRNA families (and 4 miRNAs that have not yet been classified to a known miRNA family) in the rhesus genome that expanded by SD events (Table S4, Table S5). Some RrmiRs were also duplicated by SD events in their evolutionary history. In the human genome, RdmiR genes from 4 known families and some PRdmiR genes from the mir-3179 family (hsa-mir-3179-1, hsa-mir-3179-2, hsa-mir-3179-3) expanded by SD events, in addition to the transposition effect in the past evolutionary history (Table S5). In the rhesus genome, some genes (mml-mir-3118-1, mml-mir-3118-4) from only one RdmiR family, the mir-3118 family, expanded by SD events (Table S5) and in the mouse genome, the RdmiR genes from three known miRNA gene families duplicated via SD events. We have listed some RdmiR genes from the mir-467 family and some PRdmiR genes from the mir-467 family that also expanded by SD events (Table S5). These events reflect one of the evolutionary mechanisms responsible for the expansion of miRNA families under the SD model. No common miRNA families that have been expanded by SD events were identified in all three genomes, but we found that the mir-3118 and mir-3156 families in human and rhesus expanded by SD events genome.

CNV data will also help us investigate the evolution of gene families including non-coding gene families. Some CNVs that are fixed in the population and some duplication type CNVs contain SDs [35], [36]. These SDs are often variable in copy number and can be referred to as CNVs [25]. A copy number polymorphism (CNP) refers to a CNV that occurs in more than 1% of the population [34]. The CNV data also show that duplication events have contributed to the expansion of RrmiRs. We found 85 NRdmiRs, 8 PRdmiRs and 28 RdmiRs that map to 79 locations of the human CNP data (Table S6), and 26 miRNAs (17 NRdmiRs, 3 PRdmiRs and 6 RdmiRs) that map to SD pair blocks located in CNP blocks (Table S4 and Table S6). Only one NRdmiR gene in rhesus and one RdmiR gene in mouse overlap with an SD pair and CNV block (Table S4 and Table S6). These results provide further evidence for the hypothesis that miRNA genes expanded by duplication events and indicate that the copy number of some miRNAs varies in different species.

### Functions of the common RrmiRs in the human, rhesus and mouse genomes

**Figure 7:**
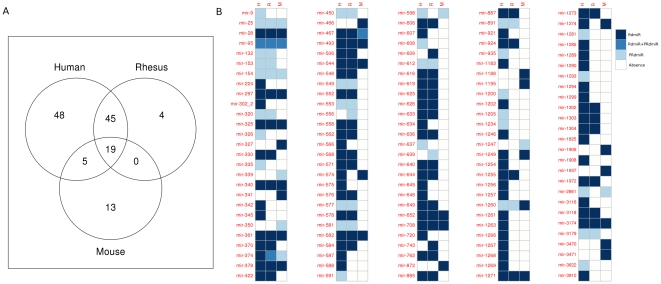
The known RrmiR families in the human, rhesus and mouse genomes. (A) Venn diagram. (B) An image of the detailed list. The miRNA families are indicated on the left of the heatmap.

Among the known miRNA families, we found 19 RrmiR families that are common to the human, rhesus and mouse genomes (Here we provisionally define hsa-mir-3174, mml-mir-3174 and mmu-mir-3174 as belonging to the mir-3174 family, because they have not yet been classified in miRBase or Rfam), 64 RrmiR families common to human and rhesus, 24 RrmiR families common to human and mouse and 19 RrmiR families common to rhesus and mouse (Figure 7, Table S2). These miRNA families are highly conserved across the species, suggesting that selective pressure may have driven the acquisition and retention of special functions.

To investigate the functions of 19 of the conserved RrmiR families, we used a computational method to classify their target gene according to their function in the cells. We found regulation of transcription, central nervous system development, and negative regulation of biological process to be the most significantly enriched GO terms in the target genes of the miRNAs from the 19 selected RrmiR families from mouse, rhesus and human (Figure S1 and Table S7). Protein complex assembly and nervous system development were the most common biological progress terms for human and rhesus, while negative regulation of cellular process and cell proliferation were the most common biological process terms for human and mouse (Figure S1 and Table S7).

**Figure 8:**
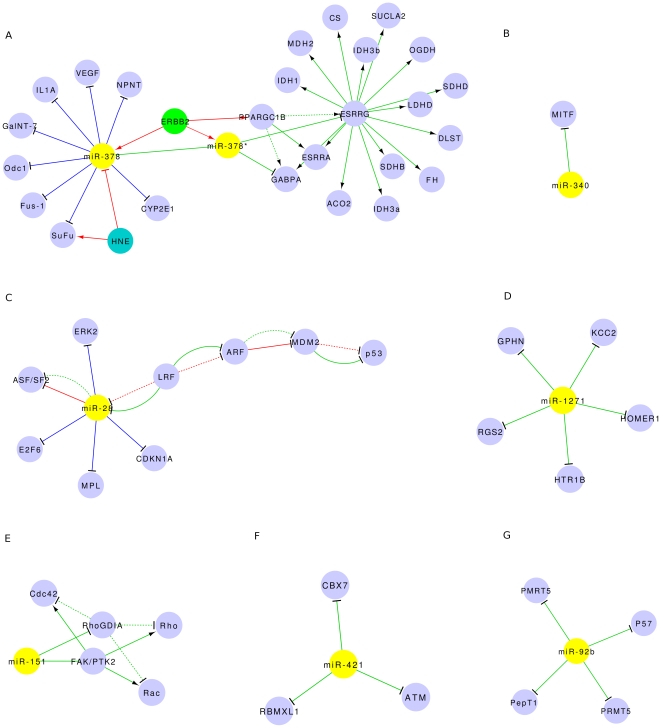
The functional networks of 7 common RrmiR families reconstructed using the Cytoscape program. (A) miR-378 (RdmiR, mir-378 family). (B) miR-340 (RdmiR, mir-340 family). (C) miR-28 (RdmiR, mir-28 family). (D) miR-1271 (RdmiR, mir-1271 family. (E) miR-151 (RdmiR, mir-28 family). (F) miR-421 (RdmiR, mir-95 family). (G) miR-92b (PRdmiR, mir-25 family).

We reconstructed functional networks by literature mining. Although very few functional studies of RrmiRs are available, experimentally validated miRNA-target interactions for quite a few RrmiRs (including miRNAs from the most common RrmiR families, Table S8) have been well documented in the miRTarBase Release 1.0 [59]. In addition, we manually curated from the literature and 17 new miRNA-target pairs for which the functions have been well studied. The functional networks of miR-92b (PRdmiR, mir-25 family, derived from GC rich tandem repeats), miR-28 (RdmiR, mir-28 family, derived from LINE), miR-151 (RdmiR, mir-28 family, derived from LINE), miR-421 (RdmiR, mir-95 family, derived from LINE), miR-1271 (RdmiR, mir-1271 family, derived from LINE), miR-340 (RdmiR, mir-340 family, derived from DNA transportable element) and miR-378 (RdmiR, mir-378 family, derived from SINE) have been reconstructed (Figure 8). In the miR-378 network (Figure 8A), ERBB2 is a transcription factor of the miR-378 gene and it's host gene PPARGC1B which encodes PGC-1β [73] and HNE, which it also appears, could downregulate miR-378 and induce the expression of its target gene, SuFu [74]. The expression of miR-378* increases during breast cancer progression and miR-378* induces the Warburg effect in breast cancer cells by inhibiting the expression of two PGC-1 partners, ERR and GABPA [73]. In the miR-28 network (Figure 8C), ASF/SF2 expression is modulated by miR-28 and miR-505 (not show here) which are negatively controlled by LRF to influence the proliferation and survival of mouse embryonic fibroblasts [75]. In Figure 8E, how miR-151 exerts this function by targeting RhoGDIA to activate Rac1, Cdc42 and Rho GTPases is shown [76]. In addition, miR-151 can function synergistically with its host gene FAK to enhance HCC cell motility and spreading [76]. Although the functions of the 7 RrmiR families displayed in Figure 8 have only been validated in human miRNAs, because they are common miRNA families in human, rhesus and mouse, they are likely to have the same functions in the other mammals. We found many RrmiRs are in cancer cells (*in vitro*) or in tumor tissues and several studies have shown that some RrmiRs are expressed in the central nervous system [77]–[80]. This experimental evidence roughly validates the functional enrichment results generated computationally.

## Discussion

As yet, little is known about the origin of most miRNAs and miRNA families in mammals. Here, we characterized miRNAs derived from repetitive elements and some miRNA families expanded by SD events in several mammalian genomes. We have found 226 RdmiRs and 52 PRdmiRs in the human genome, 115 RdmiRs and 26 PRdmiRs in the rhesus genome and 141 RdmiRs and 27 PRdmiRs in the mouse genome. Although most reports on RrmiRs have mainly concentrated on TEs, there are a few reports on miRNAs derived from tandem array sequences using computational methods and biological experiments [15], [19], [81]. In this study, we identified a number of miRNAs derived from tandem repeats and low-complexity repeats (Table S1). The most striking instance is the large mir-467 family in the mouse genome, which was derived from simple repeats. Many miRNA genes derived from repetitive elements have been identified, but the current methods used to identify miRNAs always discard the segments that map to repetitive elements annotated in the genomes. Although this may sometimes be valid, some recently identified miRNAs, such as the mmu-mir-2134 family, the mmu-mir-2135 family and hsa-mir-3172, were documented in miRBase 14 and/or miRBase 15, but because they are derived from tandem repeats (rRNA repeats, tRNA repeats), they have been removed from the more recent miRBase 16. Strictly speaking, it cannot be proven that all the potential miRNAs derived from repetitive elements are not miRNAs just because their function is, as yet, unknown. Recently, a growing body of literature (Table S8) has made it possible to validate the functions of PRdmiRs and RdmiRs, many of which were found to be expressed in tumors. We strongly suggest that small RNA fragments that map to genome regions annotated as repetitive elements should not be discarded before biological experimental data are available to verify them.

NRdmiRs, PRdmiRs and RdmiRs have some significantly different characteristics: (1) their distribution between protein-coding regions and intergenic regions is biased, (2) there are obvious differences in the MFEs of their secondary structures, and (3) because most RdmiRs are relatively young, they are relatively less conserved than NRdmiRs and PRdmiRs in vertebrates. This result agrees well with a previous report [18]. However, we did find 19 RrmiR families that were conserved in human, rhesus and mouse. We also identified many RrmiR families that are lineage-specific or that undergo is lineage-specific expansion. An example of this is the mir-467 family that is hugely expanded in mouse.

As in our previous study [20], here too we found some miRNA families (including RrmiR families) that may originate from and expand by repetitive elements. In addition, we discovered that miRNA families can also expand by SD events. Examples of this are the mir-297, mir-466, mir-467, mir-548 [16], mir-1302 [20], mir-1972, mir-3118 and mir-3179 families (which are all RrmiR families listed here) (Table S5). Our results show the complex evolutionary dynamics of some miRNAs. CNV regions may contain hundreds of genes, disease loci, functional elements and SDs. The association of CNVs with SDs has been observed in the human genome [35], [36], and Redon and his colleagues have found that nearly a quarter of the CNV regions were associated with SDs in human genome [36], while the SD-mediated non-allelic homologous recombination mechanism accounts for about a quarter (<28%) of CNVs formation [35]. Many miRNA families were produced from duplications as tandem repeats of small fragments or as large fragment segmental duplications (synonymous with copy number variation in some time). We analyzed all human miRNA family members that mapped to CNP regions and found that the distance between the loci on the same chromosome of members of one miRNA family is ∼1000-nt or more. We found 26 human miRNA genes that mapped to both SD pair blocks and CNP blocks (Table S6). In rhesus and mouse, although we found associations of SDs with miRNA genes in their genomes, only one of their miRNA genes is in both an SD pair block and a CNV block respectively (Table S6). These results provide further evidence that duplication events promoted the expansion of miRNA genes, including RrmiR genes, in the human and other primate genomes, and indicate that the copy number of some miRNAs varies in different species. In line with previous reports that most mouse duplications are distributed in discrete clusters of tandem duplications [54], we found that the miRNA genes clustered in SD blocks and were distributed mainly on chromosomes 2, 12 and 13 (Table S3), and that nearly all the miRNA genes in SD pairs tended to cluster and were located on chromosome 2 (Table S4). In mouse, although 70% of the CNV blocks were completely located in SD regions, a recent study reported that most mouse duplications are depleted of genes [54]. This could account for the finding that there are very few mouse miRNA genes associated with CNVs, and indicates a significant difference between human and mouse miRNAs. For rhesus, more evidence is needed to explain some of the reported phenomena.

In conclusion, we have presented evidence for two possible mechanisms for the origin and evolution of miRNA genes in mammals. Our main results suggest that repetitive elements contribute to the *de novo* origin of miRNAs, and that large SD events may also accelerate the expansion of miRNA families, including RdmiRs. Our work also shows how SD pair data can be used to identify miRNA paralogs. Our results indicate that some RrmiRs undergo species- or lineage-specific expansion and, while some are conserved in mammals, they are less conserved in other vertebrates compared to NRdmiRs. Moreover, we have provided both computational and experimental evidence for the functions of some common RrmiR genes that have become fixed in the three mammals studied.

## Supporting Information

Text S1 (DOC)

Figure S1 The topological GO graph for the enriched GO biology process terms of the target genes of common RrmiRs in the human, rhesus and mouse genomes. (A) human (B) rhesus (C) mouse.
(TIF)

Table S1 List of miRNA genes that overlap with repeats.
(XLS)

Table S2 List of RrmiR families from the human, rhesus and mouse genomes.
(XLS)

Table S3 List of miRNAs that overlap with segmental duplications.
(XLS)

Table S4 List of miRNAs located in segmental duplication pairs.
(XLS)

Table S5 List of miRNA families located in segmental duplications in the human, rhesus and mouse genomes.
(XLS)

Table S6 List of miRNAs locate in the copy-number polymorphism and copy-number variant blocks.
(XLS)

Table S7 The enriched GO biology process terms for the target genes of common RrmiRs.
(XLS)

Table S8 List of RrmiR-target pairs validated by biological experiments mined from various sources.
(XLS)

## References

[1] Shabalina SA, Koonin EV (2008). Origins and evolution of eukaryotic RNA
interference.. Trends Ecol Evol.

[2] Nozawa M, Miura S, Nei M (2010). Origins and evolution of microRNA genes in Drosophila
species.. Genome Biology and Evolution.

[3] Fahlgren N, Jogdeo S, Kasschau KD, Sullivan CM, Chapman EJ (2010). MicroRNA Gene Evolution in Arabidopsis lyrata and Arabidopsis
thaliana..

[4] Allen E, Xie Z, Gustafson AM, Sung GH, Spatafora JW (2004). Evolution of microRNA genes by inverted duplication of target
gene sequences in Arabidopsis thaliana.. Nat Genet.

[5] Fahlgren N, Howell MD, Kasschau KD, Chapman EJ, Sullivan CM (2007). High-throughput sequencing of Arabidopsis microRNAs: evidence for
frequent birth and death of MIRNA genes.. PLoS One.

[6] Rajagopalan R, Vaucheret H, Trejo J, Bartel DP (2006). A diverse and evolutionarily fluid set of microRNAs in
Arabidopsis thaliana.. Genes Dev.

[7] Vazquez F, Blevins T, Ailhas J, Boller T, Meins F (2008). Evolution of Arabidopsis MIR genes generates novel microRNA
classes.. Nucleic Acids Res.

[8] Tanzer A, Stadler PF (2004). Molecular evolution of a microRNA cluster.. J Mol Biol.

[9] Maher C, Stein L, Ware D (2006). Evolution of Arabidopsis microRNA families through duplication
events.. Genome Res.

[10] Zhang R, Peng Y, Wang W, Su B (2007). Rapid evolution of an X-linked microRNA cluster in
primates.. Genome Res.

[11] Jiang D, Yin C, Yu A, Zhou X, Liang W (2006). Duplication and expression analysis of multicopy miRNA gene
family members in Arabidopsis and rice.. Cell Res.

[12] Felippes FF, Schneeberger K, Dezulian T, Huson DH, Weigel D (2008). Evolution of Arabidopsis thaliana microRNAs from random
sequences.. RNA.

[13] Borchert GM, Lanier W, Davidson BL (2006). RNA polymerase III transcribes human microRNAs.. Nature Structural & Molecular Biology.

[14] Devor EJ, Peek AS, Lanier W, Samollow PB (2009). Marsupial-specific microRNAs evolved from marsupial-specific
transposable elements.. Gene.

[15] Hertel J, Lindemeyer M, Missal K, Fried C, Tanzer A (2006). The expansion of the metazoan microRNA
repertoire.. BMC Genomics.

[16] Piriyapongsa J, Jordan IK (2007). A family of human microRNA genes from miniature inverted-repeat
transposable elements.. PLoS One.

[17] Piriyapongsa J, Jordan IK (2008). Dual coding of siRNAs and miRNAs by plant transposable
elements.. RNA.

[18] Piriyapongsa J, Marino-Ramirez L, Jordan IK (2007). Origin and Evolution of Human microRNAs From Transposable
Elements.. Genetics.

[19] Smalheiser NR, Torvik VI (2005). Mammalian microRNAs derived from genomic repeats.. Trends Genet.

[20] Yuan Z, Sun X, Jiang D, Ding Y, Lu Z (2010). Origin and evolution of a placental-specific microRNA family in
the human genome.. BMC Evol Biol.

[21] Batzer MA, Deininger PL (2002). Alu repeats and human genomic diversity.. Nat Rev Genet.

[22] Bell GI (1992). Roles of repetitive sequences.. Computers & Chemistry.

[23] Meagher TR, Vassiliadis C (2005). Phenotypic impacts of repetitive DNA in flowering
plants.. New Phytol.

[24] Lander ES, Linton LM, Birren B, Nusbaum C, Zody MC (2001). Initial sequencing and analysis of the human
genome.. Nature.

[25] Feuk L, Carson AR, Scherer SW (2006). Structural variation in the human genome.. Nat Rev Genet.

[26] Biemont C, Vieira C (2006). Genetics: junk DNA as an evolutionary force.. Nature.

[27] SanMiguel P, Tikhonov A, Jin YK, Motchoulskaia N, Zakharov D (1996). Nested retrotransposons in the intergenic regions of the maize
genome.. Science.

[28] Consortium ICGS (2004). Sequence and comparative analysis of the chicken genome provide
unique perspectives on vertebrate evolution.. Nature.

[29] Bejerano G, Lowe CB, Ahituv N, King B, Siepel A (2006). A distal enhancer and an ultraconserved exon are derived from a
novel retroposon.. Nature.

[30] Nekrutenko A, Li WH (2001). Transposable elements are found in a large number of human
protein-coding genes.. Trends Genet.

[31] Ogata H, Audic S, Abergel C, Fournier PE, Claverie JM (2002). Protein Coding Palindromes Are a Unique but Recurrent Feature in
Rickettsia.. Genome Res.

[32] Cam HP, ichi Noma K, Ebina H, Levin HL, Grewal SIS (2008). Host genome surveillance for retrotransposons by
transposon-derived proteins.. Nature.

[33] Llave C, Kasschau KD, Rector MA, Carrington JC (2002). Endogenous and silencing-associated small RNAs in
plants.. Plant Cell.

[34] Scherer SW, Lee C, Birney E, Altshuler DM, Eichler EE (2007). Challenges and standards in integrating surveys of structural
variation.. Nat Genet.

[35] Kim PM, Lam HY, Urban AE, Korbel JO, Affourtit J (2008). Analysis of copy number variants and segmental duplications in
the human genome: Evidence for a change in the process of formation in
recent evolutionary history.. Genome Res.

[36] Redon R, Ishikawa S, Fitch KR, Feuk L, Perry GH (2006). Global variation in copy number in the human
genome.. Nature.

[37] Bailey JA, Yavor AM, Massa HF, Trask BJ, Eichler EE (2001). Segmental duplications: organization and impact within the
current human genome project assembly.. Genome Res.

[38] Griffiths-Jones S, Grocock RJ, van Dongen S, Bateman A, Enright AJ (2006). miRBase: microRNA sequences, targets and gene
nomenclature.. Nucleic Acids Res.

[39] Lu J, Shen Y, Wu Q, Kumar S, He B (2008). The birth and death of microRNA genes in
Drosophila.. Nat Genet.

[40] Kozomara A, Griffiths-Jones S (2010). miRBase: integrating microRNA annotation and deep-sequencing
data..

[41] Kent WJ, Sugnet CW, Furey TS, Roskin KM, Pringle TH (2002). The human genome browser at UCSC.. Genome Res.

[42] Rhead B, Karolchik D, Kuhn RM, Hinrichs AS, Zweig AS (2010). The UCSC Genome Browser database: update 2010.. Nucleic Acids Res.

[43] Schwartz S, Kent WJ, Smit A, Zhang Z, Baertsch R (2003). Human-mouse alignments with BLASTZ.. Genome Res.

[44] Jiang P, Wu H, Wang W, Ma W, Sun X (2007). MiPred: classification of real and pseudo microRNA precursors
using random forest prediction model with combined features.. Nucleic Acids Res.

[45] Smit AFA, Hubley R, Green P (1996). RepeatMasker Open-3.0.. http://www.repeatmasker.org.

[46] Karolchik D, Hinrichs AS, Furey TS, Roskin KM, Sugnet CW (2004). The UCSC Table Browser data retrieval tool.. Nucleic Acids Res.

[47] Giardine B, Riemer C, Hardison RC, Burhans R, Elnitski L (2005). Galaxy: a platform for interactive large-scale genome
analysis.. Genome Res.

[48] R Development Core Team (2009). R: A Language and Environment for Statistical
Computing..

[49] Rodriguez A, Griffiths-Jones S, Ashurst JL, Bradley A (2004). Identification of mammalian microRNA host genes and transcription
units.. Genome Res.

[50] Hinske LC, Galante PA, Kuo WP, Ohno-Machado L (2010). A potential role for intragenic miRNAs on their hosts'
interactome.. BMC Genomics.

[51] Hofacker IL, Fontana W, Stadler PF, Bonhoeffer LS, Tacker M (1994). Fast folding and comparison of RNA secondary
structures.. Monatshefte für Chemie/Chemical Monthly.

[52] Siepel A, Bejerano G, Pedersen JS, Hinrichs AS, Hou M (2005). Evolutionarily conserved elements in vertebrate, insect, worm,
and yeast genomes.. Genome Res.

[53] Blanchette M, Kent WJ, Riemer C, Elnitski L, Smit AF (2004). Aligning multiple genomic sequences with the threaded blockset
aligner.. Genome Res.

[54] She X, Cheng Z, Zöllner S, Church DM, Eichler EE (2008). Mouse segmental duplication and copy number
variation.. Nat Genet.

[55] Lee AS, Gutierrez-Arcelus M, Perry GH, Vallender EJ, Johnson WE (2008). Analysis of copy number variation in the rhesus macaque genome
identifies candidate loci for evolutionary and human disease
studies.. Hum Mol Genet.

[56] Kertesz M, Iovino N, Unnerstall U, Gaul U, Segal E (2007). The role of site accessibility in microRNA target
recognition.. Nat Genet.

[57] John B, Enright AJ, Aravin A, Tuschl T, Sander C (2004). Human MicroRNA targets.. PLoS Biol.

[58] Alexa A, Rahnenfuhrer J, Lengauer T (2006). Improved scoring of functional groups from gene expression data
by decorrelating GO graph structure.. Bioinformatics.

[59] Hsu S-D, Lin F-M, Wu W-Y, Liang C, Huang W-C (2011). miRTarBase: a database curates experimentally validated
microRNA–target interactions.. Nucleic Acids Research.

[60] Shannon P, Markiel A, Ozier O, Baliga NS, Wang JT (2003). Cytoscape: a software environment for integrated models of
biomolecular interaction networks.. Genome Res.

[61] Gibbs RA, Rogers J, Katze MG, Bumgarner R, Weinstock GM (2007). Evolutionary and biomedical insights from the rhesus macaque
genome.. Science.

[62] Waterston RH, Lindblad-Toh K, Birney E, Rogers J, Abril JF (2002). Initial sequencing and comparative analysis of the mouse
genome.. Nature.

[63] Vaz C, Ahmad HM, Sharma P, Gupta R, Kumar L (2010). Analysis of microRNA transcriptome by deep sequencing of small
RNA libraries of peripheral blood.. BMC Genomics.

[64] Altuvia Y, Landgraf P, Lithwick G, Elefant N, Pfeffer S (2005). Clustering and conservation patterns of human
microRNAs.. Nucl Acids Res.

[65] Lagos-Quintana M, Rauhut R, Lendeckel W, Tuschl T (2001). Identification of novel genes coding for small expressed
RNAs.. Science.

[66] Lagos-Quintana M, Rauhut R, Meyer J, Borkhardt A, Tuschl T (2003). New microRNAs from mouse and human.. RNA.

[67] Mourelatos Z, Dostie J, Paushkin S, Sharma A, Charroux B (2002). miRNPs: a novel class of ribonucleoproteins containing numerous
microRNAs.. Genes Dev.

[68] Dostie J, Mourelatos Z, Yang M, Sharma A, Dreyfuss G (2003). Numerous microRNPs in neuronal cells containing novel
microRNAs.. RNA.

[69] Kazazian HH (2004). Mobile elements: drivers of genome evolution.. Science.

[70] Hawkins JS, Kim H, Nason JD, Wing RA, Wendel JF (2006). Differential lineage-specific amplification of transposable
elements is responsible for genome size variation in
Gossypium.. Genome Res.

[71] Hikosaka A, Kawahara A (2004). Lineage-specific tandem repeats riding on a transposable element
of MITE in Xenopus evolution: a new mechanism for creating simple sequence
repeats.. J Mol Evol.

[72] Marino-Ramirez L, Lewis KC, Landsman D, Jordan IK (2005). Transposable elements donate lineage-specific regulatory
sequences to host genomes.. Cytogenet Genome Res.

[73] Eichner LJ, Perry MC, Dufour CR, Bertos N, Park M (2010). miR-378(*) mediates metabolic shift in breast cancer cells
via the PGC-1beta/ERRgamma transcriptional pathway.. Cell Metab.

[74] Pizzimenti S, Ferracin M, Sabbioni S, Toaldo C, Pettazzoni P (2009). MicroRNA expression changes during human leukemic HL-60 cell
differentiation induced by 4-hydroxynonenal, a product of lipid
peroxidation.. Free Radic Biol Med.

[75] Verduci L, Simili M, Rizzo M, Mercatanti A, Evangelista M (2010). MicroRNA (miRNA)-mediated Interaction between
Leukemia/Lymphoma-related Factor (LRF) and Alternative Splicing
Factor/Splicing Factor 2 (ASF/SF2) Affects Mouse Embryonic Fibroblast
Senescence and Apoptosis.. J Biol Chem.

[76] Ding J, Huang S, Wu S, Zhao Y, Liang L (2010). Gain of miR-151 on chromosome 8q24.3 facilitates tumour cell
migration and spreading through downregulating RhoGDIA.. Nat Cell Biol.

[77] Kapsimali M, Kloosterman WP, de Bruijn E, Rosa F, Plasterk RH (2007). MicroRNAs show a wide diversity of expression profiles in the
developing and mature central nervous system.. Genome Biol.

[78] He X, Zhang Q, Liu Y, Pan X (2007). Cloning and identification of novel microRNAs from rat
hippocampus.. Acta Biochim Biophys Sin (Shanghai).

[79] Nass D, Rosenwald S, Meiri E, Gilad S, Tabibian-Keissar H (2009). MiR-92b and miR-9/9* are specifically expressed in brain
primary tumors and can be used to differentiate primary from metastatic
brain tumors.. Brain Pathol.

[80] Santarelli DM, Beveridge NJ, Tooney PA, Cairns MJ (2011). Upregulation of Dicer and MicroRNA Expression in the Dorsolateral
Prefrontal Cortex Brodmann Area 46 in Schizophrenia.. Biol Psychiatry.

[81] Giraldez AJ, Mishima Y, Rihel J, Grocock RJ, Van Dongen S (2006). Zebrafish MiR-430 promotes deadenylation and clearance of
maternal mRNAs.. Science.

