# Supplementary material for: MicroRNA Genes Derived from Repetitive Elements and Expanded by Segmental Duplication Events in Mammalian Genomes
Source: PLoS One. 2011 Mar 16;6(3):e17666. doi: 10.1371/journal.pone.0017666 (PMC3059204; doi:10.1371/journal.pone.0017666)
Supplement: Text S1 — (DOC) [file pone.0017666.s001.doc]

Supplementary Files

[1. New pre-miRNAs identified in rhesus and mouse by computational method 1](#__RefHeading___Toc279915697)

[1.1 Rhesus 1](#__RefHeading___Toc279915698)

[1.2 Mouse 24](#__RefHeading___Toc279915699)

[2. pre-miRNAs predicted in SD pairs 26](#__RefHeading___Toc279915700)

[2.1 Human 26](#__RefHeading___Toc279915701)

[2.2 Mouse 28](#__RefHeading___Toc279915702)

[3. Reference 28](#__RefHeading___Toc279915703)

The following pre-miRNAs were validated by the MiPred classifier [1]. In the FASTA format sequence files, header information will include name of organism and its genome assemble version, miRNA ID, and corresponding genome coordinate. And following each sequence, there also provide its secondary structure which is in the dot-bracket notation and MFE predicted by RNAfold [2].

# 1. New pre-miRNAs identified in rhesus and mouse by computational method

## 1.1 Rhesus

>rheMac2_mml-mir-4252_chr1_9444544-9444606_-

UGGGGGGCUGGCGGCUCAUCAGUCCAGGCCAUCUGGCCACUAGGUCAGCACCAGCGCCCAAUC

..(((.(((((..(((.((((((...((((....))))))).))).))).))))).))).... (MFE:-31.10)

>rheMac2_mml-mir-1273d_chr1_13317632-13317717_+

GAAUCGCUUGAACCUGUGAGGUUGAGGCUGCACUGAGCCAAGAUCUUGCCACUGCACUUCAGCCUGGGUGAUAAGAGCGAAACUUC

...((((((..((((...(((((((((.((((.((.((.((....)))))).))))))))))))))))).....))))))...... (MFE:-32.50)

>rheMac2_mml-mir-3115_chr1_25621759-25621826_+

UCUGAAUAUGGGUUUACUAGUUGGUGGCCAAUUCAUGAGUCACCAACUAUUAGGCCUUUAUGUCCAGA

((((.(((((((((((.(((((((((((((.....)).))))))))))).)))))))..)))).)))) (MFE:-29.80)

>rheMac2_mml-mir-1976_chr1_29196140-29196191_+

GCAGCAAGGAAGGCAGGGUUCCUGAGGUGUGUCCUCCUGCCCUCCUUGCUGU

((((((((((.(((((((..((......).)..).)))))).)))))))))) (MFE:-29.80)

>rheMac2_mml-mir-3659_chr1_40968793-40968891_+

UCUACAAGCAGAUACAAGGAUGCCCUUGUACACGACACACGUGCUGCUUGUAUAGACACGAGUGUUGUCUACGAGGGCACCCUUGUGUCUGUGUGUGUG

..((((.((((((((((((.((((((((((.(((((((.(((((((......))).)))).))))))).)))))))))).)))))))))))).)))).. (MFE:-58.60)

>rheMac2_mml-mir-3671_chr1_67832814-67832901_-

UUGUUAUUGCUGCUGCUGUCACCUUUACAUGAAAGUAAAAUGUAAGUUAUUUUAUUUCUAUCAAAUAAGGACUAGUCUGCAGUGAUAU

....(((..((((.((((...((((....(((((((((((((.....))))))))))...)))...))))..))))..))))..))). (MFE:-22.40)

>rheMac2_mml-mir-320b-1_chr1_119645403-119645481_+

AAUUAAUCCCUCUCUUUCUAGUUCUUCCUAGAGUGAGGAAAAGCUGGGUUGAGAGGGCAAAUAAAUUAACUAAUUAAUU

.......(((((((..(((((((.(((((......))))).)))))))..)))))))...................... (MFE:-28.00)

>rheMac2_mml-mir-3675_chr1_123321335-123321406_-

GGAUGGUAAGUUAUGGGGCCUUCUGUAGAGAGUUCUAUGAGAACAUCUCUAAGGAACCACCAAGCUGAAUUC

((((....((((.(((((..((((.((((((((((.....)))).)))))).)))))).)))))))..)))) (MFE:-22.80)

>rheMac2_mml-mir-4258_chr1_133519334-133519419_+

ACCCCCGCCGCCACCGCCUUAGAGGCUGACUUCUUACUUUCGGUCGGUCUUCUUCCCUGGGCUUGGUGUGGGGGCGGGGGAGUGUC

.((((((((.(((((((((.(((((((((((..........)))))).))))).....))))...).)))).))))))))...... (MFE:-44.90)

>rheMac2_mml-mir-3122_chr1_158332927-158332999_-

ACCAGCUCUGUUGGGACAAGAGAACGGUCUUGUUUUGGAAGGAAGACCGUCGUUUUGUCCCAAGAGAGCUGGU

(((((((((.(((((((((((..((((((((.((.....)).))))))))..))))))))))).))))))))) (MFE:-48.30)

>rheMac2_mml-mir-1278_chr1_177463594-177463674_-

AUUUGCUCAUAGAUGAUAUGCACAGUACUCCCAGAACUCAUUAAGUUGGUAGAACCAUGCAUGUCAUCUAUGAGCGAAUAG

((((((((((((((((((((((..((.((.(((..(((.....)))))).)).))..)))))))))))))))))))))).. (MFE:-39.40)

>rheMac2_mml-mir-1255b-2_chr1_197728492-197728558_+

UCUUACGGAUAAGCAAAGAAAGUGGUUUGAGCCUCAGGAAACCACUUUCUUUGCUCAUCUGUAAGGA

((((((((((.(((((((((((((((((..........))))))))))))))))).)))))))))). (MFE:-37.20)

>rheMac2_mml-mir-3119-1_chr1_199850264-199850348_-

AUUAACUCUAGCUUUUAAUUUUGAUGGCAAAGGUGUAGCUAAACAAUCUAUGUCUUUGCCAUCAAAGUUAAAAGCCAGAGUUAAU

(((((((((.(((((((((((((((((((((((..(((.........)))..))))))))))))))))))))))).))))))))) (MFE:-47.10)

>rheMac2_mml-mir-1295_chr1_200795070-200795148_-

AGGACAUUUUACCCAGAUCCGUGGCCUAAUCAGAAAUGUGGCCUGUGAUUAGGCCACAGAUCUGGGUGAAAUGUCCUCC

(((((((((((((((((((.((((((((((((.(.........).)))))))))))).))))))))))))))))))).. (MFE:-54.10)

>rheMac2_mml-mir-3123_chr1_215908342-215908396_-

AUGGAUUUGAUUGAAUGAUUCUCCGGAGCCCAGAGAACUGUUUAAUCAUGUAUCC

..((((.(((((((((..(((((.((...)).)))))..)))))))))...)))) (MFE:-19.20)

>rheMac2_mml-mir-1537_chr1_221164716-221164765_+

ACAGCUGUAAUUAGUCCUGUCCACAGGGAAAACUGUCUAAUUACAGUUGU

(((((((((((((((((((....)))))........)))))))))))))) (MFE:-21.00)

>rheMac2_mml-mir-3921_chr2_20165154-20165229_-

CCUAGCCCAGUACAAGGCAUAUGGUACCCCUAAGUCUCUGAGUACCAUAUGCCUUGUACUGGGCUAGGUAACAUGG

(((((((((((((((((((((((((((..............)))))))))))))))))))))))))))........ (MFE:-51.94)

>rheMac2_mml-mir-544b_chr2_44735778-44735856_+

GGAAUUUUGUUAAAAUACAGAAUCCAGUUCUGUAGCUCUUGAGACUGGACCUGGGGUUGUGCAUUUCUAACAAGGUUCC

((((((((((((((((.((.(((((.((((.((..(....)..)).))))...))))).)).)))).)))))))))))) (MFE:-26.90)

>rheMac2_mml-mir-378b_chr2_50695565-50695621_-

GGUCAUUGAGUCUUCAAGGCUAGUGGAAAGAGCACUGGACUUGAAGGCAGAAGGACC

((((.....(((((((((.((((((.......)))))).))))))))).....)))) (MFE:-25.60)

>rheMac2_mml-mir-1284_chr2_64673119-64673238_+

AUUUUGAUAUAUAAGCCAGUUUAAUGUUUUCUGUACAGACCCUGGCUUUUCUUAAAUUUUAUAUAUUGGAAAGCCCAUGUUUGUAUUGGAAACUGCUGGUUUCUUUCAUAUUGAAAAUCU

.(((..((((..((((((((.....((((((.((((((((...((((((((................))))))))...)))))))).)))))).)))))))).....))))..))).... (MFE:-37.59)

>rheMac2_mml-mir-3136_chr2_67104168-67104245_+

AAUAUGAAACUGACUGAAUAGGUAGGGUAAUUUUUCAGUGACUGCACAUGGCCCUACAUAUUCAGUCAGUUUCAUAUU

((((((((((((((((((((.(((((((.......(((...)))......))))))).)))))))))))))))))))) (MFE:-40.32)

>rheMac2_mml-mir-3938_chr2_80519795-80519895_+

AGGAAUUUUUAACCUGAUCACCAGAUUAUCUACAAGGGAAUUUUUUUAAUUUUUAAAAUUCCCUUAUGGAUAAUCUGGUGAUCAGGUUAGAUGGCUCCAUG

.(((...(((((((((((((((((((((((((.(((((((((((..........))))))))))).)))))))))))))))))))))))))....)))... (MFE:-57.30)

>rheMac2_mml-mir-4271_chr2_87175054-87175120_-

AAAUCUCUCUCCAUAUCUUUCCUGCAGCCCCCAGGUUGGGGGGAAGAAAAGGUGGGGAAUUAGAUUC

.(((((.(((((((.((((((((.(((((....))))).))))))))....)))))))...))))). (MFE:-30.00)

>rheMac2_mml-mir-1263_chr2_123318344-123318452_+

UUACCCCAAAAUAUGGCACUACGGCAUACUGAGUGUGUCAGUAUUAAAAUAUUCAGUAUGCCAGUAUUAAAAUACUCAGUGUGCCAGGGUACCAUAUUUUGGAGUAGCA

((((.(((((((((((.(((..((((((((((((((...((((((...((((...))))...))))))...))))))))))))))..))).))))))))))).)))).. (MFE:-47.70)

>rheMac2_mml-mir-3134_chr2_158033357-158033430_-

UGUAUCCAAUGUGUAGUCUUUUAUCCCUCACAUGGAGUAAAAUAUGAGGGAUAAAAGACUACAUAUUGGGUGCA

.(((((((((((((((((((((((((((((.((........)).))))))))))))))))))))))))))))). (MFE:-48.20)

>rheMac2_mml-mir-3714_chr2_159246637-159246701_+

GAAGGCAGCAGUGCUCCCCUGCGACAUGCUCUGUCACUGGGCAGGGAAGACGCUGCUGCUACGUC

...((((((((((((.((((((((((.....)))).....)))))).)).))))))))))..... (MFE:-33.90)

>rheMac2_mml-mir-1248_chr2_179144219-179144324_+

UUUACCUUCUUGUAUAAGCACUGUGCUAAAAUUGCAGACACUAGGACUAUGUCUUGGUUUUUGCAAUAAUGCUAGCAGAGUACACACAAGAAGAAAAGUAACAGCA

.....((((((((....(.(((.(((((..((((((((.((((((((...)))))))).)))))))).....))))).))).)..))))))))............. (MFE:-34.50)

>rheMac2_mml-mir-548x_chr3_28243706-28243779_+

AGGUUAGUGUAAAAGUAAUUGCAGUUUUUGCAUUACUUCCAAUGGUAAAAAAACAAUUACUUUCACACCAAUCU

(((((.((((.((((((((((...((((((((((......))).)))))))..)))))))))).)))).))))) (MFE:-21.90)

>rheMac2_mml-mir-3914-1_chr3_56508230-56508327_-

UGGACUUCAGAUUUAACUUUUCAUUUUCUGAUUCCUUCUAAUGAGUAUGCUUAACUUGGUAGAAGGAACCAGAAAAUGGAAGUUGAGUAGGAACUCUA

((((.(((..((((((((((.(((((((((.(((((((((..((((.......))))..))))))))).)))))))))))))))))))..))).)))) (MFE:-41.20)

>rheMac2_mml-mir-3914-2_chr3_56508232-56508325_+

GAGUUCCUACUCAACUUCCAUUUUCUGGUUCCUUCUACCAAGUUAAGCAUACUCAUUAGAAGGAAUCAGAAAAUGAAAAGUUAAAUCUGAAGUC

((.(((......(((((.(((((((((((((((((((...(((.......)))...)))))))))))))))))))..)))))......))).)) (MFE:-32.30)

>rheMac2_mml-mir-548n_chr3_91232094-91232164_+

AGGUUGGCGCAAAAGUAAUUACAGGUUUUGUCAAAAUAGCAAAACACGCAAUUACUUUUGCAGCAACCUAA

((((((..((((((((((((.(..(((((((.......)))))))..).))))))))))))..)))))).. (MFE:-28.20)

>rheMac2_mml-mir-3146_chr3_106443246-106443324_+

GCUAAGUCCUUUCUUUCUAUCCUAGUAUAACUUGAAGAAUUCAAAUAGUCAUGCUAGAACAGAAAGAAUGGGAGUUGGC

(((((.((((((((((((...(((((((.((((((.....)))...))).)))))))...)))))))).)))).))))) (MFE:-29.10)

>rheMac2_mml-mir-3666_chr3_151996873-151996983_+

AGUAAGGUCCGUCAGUUGUAAUGAGACCCAGUGCAAGUGUAGAUGCCGACUCCGUGGCAGAGUUCAGCGUUUCACACUGCCUGGUCUCUGUCACUCUAUUGAAUUAGAUUG

.....((((..(((((.((.((((((((.((.(((.((((((((((.(((((.......)))))..)))))).))))))))))))))).)).))...)))))....)))). (MFE:-34.50)

>rheMac2_mml-mir-548f-4_chr3_184679371-184679475_-

GAGUUCUAAUGUAUUAGGUUGGUGCAAAAGUAAUAGUGGUUUUUGCCAUUAAAAGUAAUGACAAAAACCAUAAUUACUUUUGGAACAAUAUUAAGAGAAUUUCAG

(((((((....((....((((.(.((((((((((.((((((((((.(((((....))))).)))))))))).)))))))))).).))))..))..)))))))... (MFE:-34.20)

>rheMac2_mml-mir-3143_chr4_26914260-26914328_+

UAGAUCACAUUGUAAAGCGCUUCUUUCCCGGUUGGGCUGGAGUAACUCUUUACAAUGUUUUUUGAUGUA

((((..(((((((((((.(((((...(((....)))..)))))....)))))))))))..))))..... (MFE:-21.20)

>rheMac2_mml-mir-3934_chr4_33409348-33409448_+

CACAGGCCUGUCUCAUUUUCAGGUGUGGAAACUGAGGCAGGAGGUGGUGAAGUAACUUGCUCAGUUUGCACAGCUGGGAAGUGGAGCAGGGAUUUGAAUCC

......(((((..(((((((((.((((.((((((((.((((..............)))))))))))).)))).)).)))))))..)))))........... (MFE:-40.54)

>rheMac2_mml-mir-3925_chr4_36382303-36382379_-

GUGGGAAUAGCAAGAGAACUAAAAUUAGAGCUUGUCACAUCUCCAGACUCCACUUUUAGUUCUCUUGCUAUUUCCAC

((((((((((((((((((((((((...(((((.(........).)).)))...)))))))))))))))))))))))) (MFE:-38.70)

>rheMac2_mml-mir-548u_chr4_57002217-57002298_+

AUUAGGAUGGUGCAAAAGUAAUUGUGGUUUUUGUCUUUACUUUUAAUGGCAAAGACUGCAAUUACUUUUGCACCAACCCAAU

....((.(((((((((((((((((..(((((((((.(((....))).)))))))))..))))))))))))))))).)).... (MFE:-43.80)

>rheMac2_mml-mir-4282_chr4_69211784-69211850_-

GGUGAAGUUCCAGGGGAAGAUUUUAGUAUGCCACAUUUCUAAAAUUUGCAUCCAGGAACAUCAUCCU

(((((.(((((.(((..(((((((((.(((...)))..)))))))))...))).))))).))))).. (MFE:-22.70)

>rheMac2_mml-mir-2113_chr4_93966486-93966576_+

UUUUCAAAGCAAUGUGUGACAGGUACAGGGACAAAUCCCGUUAAUAAGUAAGAGGAUUUGUGCUUGGCUCUGUCACAUGCCACUUUGAAAA

.((((((((...(((((((((((..((((.((((((((..(((.....)))..)))))))).))))..)))))))))))...)))))))). (MFE:-39.40)

>rheMac2_mml-mir-1273c_chr4_108119433-108119518_-

UCCAGCCUGGGUGACAAAAUGAGACCCUGUCUUUUUUUUUUUUUUUUUUUUUUUUGAGACAGUCUCGCUCUGUUGCGCAAGCUGGA

((((((.((.(..(((.(.((((((..((((((......................)))))))))))).).)))..).)).)))))) (MFE:-31.05)

>rheMac2_mml-mir-3668_chr4_123566764-123566838_-

AUAUAUGAACUGUAGAGAUUGAUCAAAAUAGAUUUUAUCAAAAUAGUUUUGAGCAUUCUCCACAAUUUUAUGUAU

(((((((((.(((.((((.((.(((((((..((((.....)))).))))))).)).)))).)))..))))))))) (MFE:-17.90)

>rheMac2_mml-mir-3145_chr4_125407858-125407938_+

AUAUAAGGUCAACUCCAAACAUUCAAAACGCAUUGUUGAAUGGAAUUAGGUAUUUUGAGUGUUUGGAAUUGAACUUGUAUG

(((((((.((((.(((((((((((((((..(...(((......)))...)..))))))))))))))).)))).))))))). (MFE:-31.70)

>rheMac2_mml-mir-3138_chr5_4964414-4964494_-

CCUCCCUUGGCACUUCCCCCCACCUCACUGUCCGGGUACCCACAAGACUAGACAGUGAAGUAAUGAGAGUACCGAGGAGGG

(((((.((((.(((..(....((.((((((((..(............)..)))))))).))...)..))).))))))))). (MFE:-31.30)

>rheMac2_mml-mir-1269_chr5_63114179-63114283_-

UGGAUUCCCUAGACCAGGAAAGCCAGUUGGUGUGGCUCAGUCCAAGUCUGAGCACGUGAGCAAUGCCUGGACUGAGCCACGCUACUGGCUUACCUGGUCUCCAGC

((((.......(((((((.((((((((.((((((((((((((((.((....((......))...)).)))))))))))))))))))))))).))))))))))).. (MFE:-63.81)

>rheMac2_mml-mir-3684_chr5_91904314-91904387_+

AAUCUAAAGGAUCUGUGCUAGGUUUAACAUGUUGAGCAUUACUCAUGUUAAACCUAGUACAGGUCCUUUAGAUU

(((((((((((((((((((((((((((((((............))))))))))))))))))))))))))))))) (MFE:-47.00)

>rheMac2_mml-mir-1255a_chr5_94298263-94298361_-

GAGUUGCUUCUCAAGGAUGAGCAAAGGAAGUAGUUUUUUUAGAUUCCAAAGAAACUACCUACUUUGCUCAUCUUUGAGAAGCAACUCUUUAUCCAUUAA

((((((((((((((((((((((((((...(((((((((((.......)))))))))))...))))))))))))))))))))))))))............ (MFE:-56.20)

>rheMac2_mml-mir-3139_chr5_135765429-135765504_+

GGCUCAGAGUAGGAGCUCAACAGAUGCCUGUUGACUGAAUAAUAAACAAGUAUCGCAGGAGCUUUUGUUAUGUGCC

(((.((...(((((((((....(((((.((((............)))).)))))....)))))))))...)).))) (MFE:-25.90)

>rheMac2_mml-mir-548g_chr5_139545660-139545747_-

AGUUAUUAGAUUGGUGCAAAAGUAAUUUCUGUUUUUGGUUACUUUCUAUAGCAAAACCGUAAUUACUCUUGUACCAACAUAAUACUUC

...(((((..(((((((((.(((((((.(.((((...((((.......)))).)))).).))))))).)))))))))..))))).... (MFE:-24.10)

>rheMac2_mml-mir-3140_chr5_144708317-144708404_-

CCUCUUGAGGUACCUGAAUUACCAAAAGCUAUGUAUUCUGAAGUUAUUGAAAAUAAGAGCUUUUGGGAAUUCAGGUAGUUCAGGAGUG

.((((((((.((((((((((.(((((((((...((((.(.((....)).).))))..))))))))).)))))))))).)))))))).. (MFE:-41.40)

>rheMac2_mml-mir-3688_chr5_151294958-151295050_-

UCUUCACUUUCAAAAGUGACAAAGUCUUUCCAUAUGUAUGUAUGUGUGUCUAUUACACAUAUGGAAAGACUUUGCUACUCUUUAAAGUGAAGA

((((((((((.((.((((.((((((((((((((((((..(((...........))))))))))))))))))))).)))).)).)))))))))) (MFE:-42.00)

>rheMac2_mml-mir-548t_chr5_165321658-165321732_+

AGGUUGGUGCAAAAGUGAUCACGGCUUUUGCGAUUUUUUUAAUGACAAAAACCGCAAUUACUUUUGCACAAACCU

(((((.(((((((((((((..(((.(((((..(((.....)))..))))).)))..))))))))))))).))))) (MFE:-31.10)

>rheMac2_mml-mir-1305_chr5_174283415-174283500_+

AAGAUCCUGCAGUUUCUACCAUCAGUUUUGAACAUUUAUUGUAAAGAUACUUUUAAACACUAAUGGGAGAGACAGCAGGAUUCUCC

..((((((((.((((((.((((.(((((((((....((((.....))))..)))))).))).)))).)))))).)))))))).... (MFE:-27.70)

>rheMac2_mml-mir-4277_chr6_1651959-1652039_-

UUGGGUCGAGGCUGUUCUGAGCACAGAGCUUGGUGCCGCCACUGCCCGGUGCCCUGCUCAGCUCGAGUCCUUGUACCCCUC

..(((((((((((....(((((...((((..((..(((........)))..))..)))).))))))).))))).))))... (MFE:-36.40)

>rheMac2_mml-mir-4280_chr6_83356002-83356077_-

AAUCAGGGUGGAGCGUAGUUCUGAGCAGAGCCUUAAAGGAUGAGGUAUGUCCAAGACUGAAUGACACCUUUGUGAU

.(((((((((..(..((((..((..((..((((((.....)))))).))..))..))))..)..)))))...)))) (MFE:-24.60)

>rheMac2_mml-mir-3660_chr6_86281957-86282056_-

GAAAGAAGAACUGGACAAAAUUAAAAUGCUCUUCUGUCAUGGUAAUAGUUCAUAUGGGCACUGACAGGAGAGCAUUUUAACUUUGUCAAGUGUGUCUGCU

...(((...(((.((((((.(((((((((((((((((((..((.(((.....)))..))..))))))))))))))))))).)))))).)))...)))... (MFE:-42.40)

>rheMac2_mml-mir-548p_chr6_97084765-97084848_-

AUUAGGUUGGUAUAAAAUUAAUUGCAGUUUUUGCCAUUACUUUCAAUAGCAAAAACCACAAUUACUUUUGCACCAAUGUUAUAC

.....((((((.(((((.((((((..((((((((.(((......))).))))))))..)))))).))))).))))))....... (MFE:-23.20)

>rheMac2_mml-mir-3661_chr6_130632545-130632640_+

CACCUUCUCGCAGAGGCUCUUGACCUGGGACUCGGACAGCUGCUUGCACUCGUUCAGCUGCUCGAUCCACUGGUCCAGCUCCUUGGUGAACACCUU

.......((((.((((..((.((((..(((.(((..((((((...((....)).))))))..))))))...)))).))..)))).))))....... (MFE:-36.40)

>rheMac2_mml-mir-3655_chr6_137048912-137048994_+

GCUUGUCGCUGUGGUGUUGCAGUUGGAGACUUGAUUGUUGGUGACAGCGAAACAACGAUAACAAAAUGCCAGAGCGAGAUAGU

((((.(((((.(((((((...((((..(..(((.((((((....))))))..))))..))))..))))))).))))).).))) (MFE:-28.80)

>rheMac2_mml-mir-1303_chr6_151049655-151049744_+

GGCUGGGCAACAUAGUAAGACCUCAACUCUACAAUUUUUUUUUUUUUUUUUUAAUUUUAGAGACAGAGUCCUGCUAUGUUGCCAGGCUUC

((((.((((((((((((.(((((...(((((.((((................)))).)))))..)).))).)))))))))))).)))).. (MFE:-33.09)

>rheMac2_mml-mir-3912_chr6_167769206-167769310_-

AAAGAGGAAUGAACAGUUAAAUUAUAACAUAUCCAUAAUAUGCGUUAGUUGUGGACACAUACUAACGCAUAAUAUGGAUAUGUUAUAAUUUAACUCUUCCUUUCU

....(((((.(((.(((((((((((((((((((((((.(((((((((((.(((....)))))))))))))).))))))))))))))))))))))).))).))))) (MFE:-53.90)

>rheMac2_mml-mir-1271_chr6_172840489-172840574_+

CACCCAGAUCAGUGCUUGGCACCUAGCAAGCACUCAGUAAAUACUUGUUGAGUGCCUGCUAUGUGCCGGGCAUUGUGCUGAGGGCU

..(((((..(((((((((((((.(((((.(((((((((((....))))))))))).))))).)))))))))))))..))).))... (MFE:-49.60)

>rheMac2_mml-mir-3942_chr7_13719120-13719228_-

UCUUCGGUAUGAUACCUCAAAGAAGUAAUACUGUUAUCUGAAAUAGGCUGUGAAGAUAACAGUAUUUUAGAUAACAGUAUUACAUCAUUGAAGUGUCAUAUUCACUGAC

...((((((((((((.((((.((.((((((((((((((((((((..(((((.......))))))))))))))))))))))))).)).)))).)))))))....))))). (MFE:-47.40)

>rheMac2_mml-mir-1282_chr7_22186469-22186569_-

CCUUCUUCUCGUUUGCCUUUUUCUGCUUCUGCUGCAUGAUCUCCGAGUCCCUGGGGGUAGAGAUGAUGGGGCACUGGGAGGUACCAGAGGGCAAAAAGGAC

......(((..((((((((((..((((((((((.(((.(((((....(((....)))..))))).))).)))....)))))))..))))))))))..))). (MFE:-39.40)

>rheMac2_mml-mir-1266_chr7_30580128-30580211_-

ACAGAUAGUGUCCCUCAGGGCUGUAGAACAGGACUGGGAUUACCGAAGCCCUGUUCUAUGCCCUGAGGGACACUGAGCAUGUCA

.....((((((((((((((((.((((((((((.((.((....))..)).))))))))))))))))))))))))))......... (MFE:-52.20)

>rheMac2_mml-mir-2116_chr7_37542190-37542269_-

GACCUAGGCUAGAAGUUGUUAGCAUGGGAGGUUUUCCCAUGCUAAGAAGUCCUCCCAUGCUAAGAACUCCUAGACUAGGA

..(((((.((((.((((.(((((((((((((.((((.........)))).))))))))))))).)))).)))).))))). (MFE:-44.80)

>rheMac2_mml-mir-1179_chr7_68216167-68216257_+

GGCUGGAAAGGAAGAAGCAUUCUUUCAUUGGUUGGUGUGUAUUGCCUUGUCAACCAAUAAGAGGAUGCCAUUUAUCCUUUUCUGACUAGCU

((((((((((((.((((((((((((.((((((((..(..........)..)))))))).)))))))))..))).)))))).....)))))) (MFE:-33.60)

>rheMac2_mml-mir-3174_chr7_69654319-69654405_+

AUUACCUGGUAGUGAGUUAGAAAUGCAGAGCCCCGGGCUUCUCAGCAAACCUACUGGAUCUGCAUUUUAAUUCACAUGCAUGGUAAU

((((((..((((((((((((((.((((((...(((((((....))).......)))).))))))))))))))))).)))..)))))) (MFE:-31.81)

>rheMac2_mml-mir-3171_chr7_90519278-90519349_-

UAUAUAUAGAUGUAUGGAAUCUGUAUAUAUCUAUAUGUAUGUGUAUAUAAAUAUUCCAUAAAUCUAUAUAUG

(((((((((((.((((((((...(((((((.(((....))).)))))))...)))))))).))))))))))) (MFE:-24.30)

>rheMac2_mml-mir-548h-1_chr7_127115289-127115389_-

UCUGCCUAUUAGGUGGGUGCCAAAGUAAUCAUGGUUUUUGUCAUUACUUUCAAUGGCAAAACCGGAAUUACUUUUGCACUGACCUAAUAUUAAACCAGAUA

((((..((((((((.(((((.((((((((..(((((((.((((((......)))))))))))))..)))))))).))))).))))))))......)))).. (MFE:-42.20)

>rheMac2_mml-mir-3173_chr7_158377870-158377937_-

UCCCUGCCCUGCCUGUUUUCUUCUUUGUGGUUUUAUGAAAGCGAAGGAGGAAACAGGCAGGCCAGGGA

((((((.(((((((((((((((((((((..((....))..))))))))))))))))))))).)))))) (MFE:-46.60)

>rheMac2_mml-mir-1197_chr7_164311358-164311445_+

ACUUCCUGGUAUUUGAAGAUGCGGUUGACCAUGGUGUGUACGCUUUAUUUAUAACGUAGGACACAUGGUCUACUUCUUCUCAAUAUCA

.......((((((.(((((...(((.(((((((...(.((((............)))).)...))))))).))))))))..)))))). (MFE:-25.60)

>rheMac2_mml-mir-1193_chr7_164314983-164315060_+

GUAGCUGAGGGGAUGGUAGACCGGUGACGUGCACUUCAUUUACGAUGUAGGUCACCCGUUUGACUAUCCACCAGCGCC

...((((.(.(((((((((((.((((((.((((..((......)))))).)))))).)))).))))))).)))))... (MFE:-35.40)

>rheMac2_mml-mir-1185-1_chr7_164327472-164327557_+

UUUGGUACUUGAAGAGAGGAUACCCUUUGUAUGUUCACUUGAUUAAUGGUGAAUAUACAGGGGGAGACUCUUAUUUGCGUAUCAAA

.(((((((.(((((((((..(.(((((((((((((((((........))))))))))))))))))..))))).)))).))))))). (MFE:-38.90)

>rheMac2_mml-mir-1185-2_chr7_164328692-164328777_+

UUUGGUACUUGAAGAGAGGAUACCCUUUGUAUGUUCACUUGAUUAAUGGUGAAUAUACAGGGGGAGACUCUUAUUUGCGUAUCAAA

.(((((((.(((((((((..(.(((((((((((((((((........))))))))))))))))))..))))).)))).))))))). (MFE:-38.90)

>rheMac2_mml-mir-548v_chr8_17559922-17560001_-

AAUACUAGGUCGGUGCAAAAGUAAUUGAGGUUUUGCCAUCGUGCCAAAAGCCGCAAUUACUUUUGCACCAACCUAAUAUU

((((.(((((.((((((((((((((((.((((((((......))..)))))).)))))))))))))))).))))).)))) (MFE:-39.10)

>rheMac2_mml-mir-4288_chr8_28652432-28652500_-

AUGGAGGUGGAGAGAGUCAUCAGCAGCACUGAACAGGCUGUGUUGUCUGCUGAAUUUCCACGUCAUUUG

...((.(((((((...(((.(((((((((..........)))))).))).))).))))))).))..... (MFE:-27.30)

>rheMac2_mml-mir-3148_chr8_30186090-30186166_-

AAGUUAAGAUGGAGAAAACUGAUGUAUGCUUACUGAUGUGGCCAACAAGCAUACAUCAAGUUUUUUCAACUCAACUC

.((((.((...((((((((((((((((((((..((.......))..))))))))))).)))))))))..)).)))). (MFE:-27.70)

>rheMac2_mml-mir-3118-4_chr8_44496442-44496516_-

CACACUACAAUAAUUUUCAUAAUGCCAUCACACACAAUCACCAUGUGACUGCAUUAUGAAAAUUCUUGUAGUGUG

((((((((((.((((((((((((((..(((((...........)))))..)))))))))))))).)))))))))) (MFE:-35.10)

>rheMac2_mml-mir-3149_chr8_79439300-79439368_-

AUACACACAUGUACACACACGUCACCCACAGACAUAUAUACAUAUGUUUUUAUGGAUAUGUGUGUGUAU

..........((((((((((((...(((.((((((((....))))))))...)))..)))))))))))) (MFE:-23.00)

>rheMac2_mml-mir-3150b_chr8_97653543-97653629_-

CAGGGAAAGCAGGCCAACGUCCAGGAUCUCCCCAGCCUUGGCGUUCAGAUGCUGAGAAGAUCCUCAAGGUUGGCCUGCUUUCCCCUC

..((((((((((((((((.(..(((((((.(.((((.((........)).)))).).)))))))..).))))))))))))))))... (MFE:-50.40)

>rheMac2_mml-mir-3150_chr8_97653546-97653626_+

GGGAAAGCAGGCCAACCUUGAGGAUCUUCUCAGCAUCUGAACGCCAAGGCUGGGGAGAUCCUGGACGUUGGCCUGCUUUCC

.(((((((((((((((.((.((((((((((((((...((.....))..)))))))))))))).)).))))))))))))))) (MFE:-56.50)

>rheMac2_mml-mir-1273_chr8_102535456-102535556_-

UGAGGCAGGAGAAGUGCUUGAACCCGGGAGGUGGAGGUUGCAGUGAGCCAAGAUCGUGCCACUGCACUCCAGCCUGGGCAACAGAGCGACUCUUUCUUGGA

.....((((((((((((((...((((((...(((((..(((((((.(((......).)))))))))))))).)))))).....)))).))).))))))).. (MFE:-44.40)

>rheMac2_mml-mir-3151_chr8_105677241-105677317_+

GGGGUGACGGGUGGCGCAAUGGGAUCAGGUGCCUCAAAGGGCAUCACAUCUGAUCCCACAGCCUCACCCAUCACCCC

(((((((.((((((.((..(((((((((((((((....)))))).....)))))))))..)).)))))).))))))) (MFE:-49.60)

>rheMac2_mml-mir-2053_chr8_115095732-115095822_+

CUUGCCAUGUAAAUACGGAUUUAAUUAACACUUGCAACCUGUGAAGAUGCAAAACUUUAAGUGUUAAUUAAACCUCUAUUUACAUAGCAAG

(((((.(((((((((.((.(((((((((((((((.....(((......)))......))))))))))))))).)).))))))))).))))) (MFE:-31.70)

>rheMac2_mml-mir-3610_chr8_119391102-119391174_-

AAGAGCCGCGGCGUAACGGCAGCCAUCUUGUUUGUUUGAGUGAAUCGGAAAGGAGGCGCCGGCUGUGGCGGCG

....(((((.(((...((((.(((.((((.....(((((.....))))).)))))))))))..))).))))). (MFE:-33.10)

>rheMac2_mml-mir-1204_chr8_130408897-130408963_+

ACCUCGUGGCCUGGUCUCCAUUAUUUGAGAUGAGUUACAUCUUGGAGGUGAGGACGUGCCUCGUGGU

(((.((.(((...(((..((((.((..(((((.....)))))..))))))..)))..))).)).))) (MFE:-26.80)

>rheMac2_mml-mir-3686_chr8_132074430-132074515_-

CUCACCUCAUUCAUUUACCUUCUCUUAACGAUCACUUUUCUGCAUCGGACAGUGAUCUGUAAGAGAAAGUAAAUGAAUGAGGUGAG

((((((((((((((((((.((((((((..(((((((.(((......))).)))))))..)))))))).)))))))))))))))))) (MFE:-50.20)

>rheMac2_mml-mir-1302-7_chr8_144301426-144301496_-

ACAACAUUUUUUAGUACAUGUAUGUCUGGUGCAAUCAUUGGGACAUACUUAUGCUAAAAAAAUUAGUAUUC

......((((((((((...(((((((..(((....)))..).))))))...)))))))))).......... (MFE:-19.40)

>rheMac2_mml-mir-3155_chr9_6438073-6438154_+

UCCGGGCAUCAGCUCCCACUGCAGAGGCUGGGGAGCCGGACAGCUCCCUUCCCAAGCUCUGCAGUGGGAACUGAUGCCUGGA

((((((((((((.(((((((((((((..(((((((..(((....))))))))))..))))))))))))).)))))))))))) (MFE:-63.20)

>rheMac2_mml-mir-1265_chr9_14718384-14718469_+

ACGGUUUGGGACUCAGGAUGUGGUCAAGUGUUGUCAAGGCAUGUUCGGUGAUAAUACUUGACCACAUUUUGAAUCCCAAACCACAU

..(((((((((.((((((((((((((((((((((((.((.....))..)))))))))))))))))))))))).))))))))).... (MFE:-53.40)

>rheMac2_mml-mir-1915_chr9_21707371-21707450_-

UGAGAGGGCGCGCCUUGCCUCGUUGCCCGGGCAGUGCACCCGUGGGCCCCAGGGCGACGCGGCGGGGGCGGCCCUUGCGA

.(..(((((((.(((((((.(((((((((((..((.((....)).))))).)))))))).))))))))).)))))..).. (MFE:-50.80)

>rheMac2_mml-mir-3611_chr9_35294421-35294505_-

AACAGGUCUAGUAAGAAUUUUUUUCUUUCUUCACAAUUAUGAAGGAAAAGAAAUUGUGAAGAAAGACAUUCUUACUAGUUUUGCU

..((((.(((((((((((.....(((((((((((((((.............))))))))))))))).))))))))))).)))).. (MFE:-31.12)

>rheMac2_mml-mir-3156-2_chr9_37577953-37578031_-

UGCAGAAGAAAGAUCUGGAAGUGGGAGAAAUUUUCACUGUAUAUAGUGACUUCCACUUCCAGAUCUUUCUCUCUGUGUA

.((((((((((((((((((((((((((......((((((....))))))))))))))))))))))))))).)))))... (MFE:-45.90)

>rheMac2_mml-mir-3156-1_chr9_45406850-45406924_-

GCAGAAGAAAGAUCCGGAAGUGGGAGAAACUUUCACUAUAUACAGUGGCUCCCACUUCCAGAUCUUUCUCUCUGU

((((((((((((((.(((((((((((..(((............)))..))))))))))).))))))))).))))) (MFE:-41.70)

>rheMac2_mml-mir-606_chr9_61458975-61459056_-

UGUGUCUUUUGUUUUUAGUAGUUGUGCCACCCACCCCAUCAUAGUAAAACUACUAAAAACCAAAGAUACAAGUGCCUAACCA

(((((((((.(((((((((((((.(((................))).))))))))))))).)))))))))............ (MFE:-25.89)

>rheMac2_mml-mir-1296_chr9_73887076-73887167_+

ACCUACCUAACUGGGUUAGGGCCCUGGCUCCAUCUCCUUUAGGAAAACCUUCUGUGGGGAGUGGGGCUUCGACCCUAACCCAGGUGGGCUGU

.(((((((....(((((((((.(..((((((((..(((.(((((.....))))).)))..))))))))..).)))))))))))))))).... (MFE:-50.20)

>rheMac2_mml-mir-3924_chr9_80031369-80031447_+

UAAAAGUAGUAGUCAAAUAUGCAGAUCUAUGUCAUAUAUACAGAUAUAUAUAUAUAUGUGACUACUACUUUUUUGUUUA

.((((((((((((((.(((((...((.((((((.........)))))).)).))))).))))))))))))))....... (MFE:-24.00)

>rheMac2_mml-mir-3157_chr9_95601134-95601219_-

GGGAAGGGCUUCAGCCAGGCUACUGCGGUCUGCUUUGUGCCAACACUAGGGGUGAUGACUGCCCUAGUCUAGCUGAAGCUUUUCCC

(((((((((((((((.((((((..((((((....(..(.((.......)).)..).))))))..)))))).))))))))))))))) (MFE:-47.40)

>rheMac2_mml-mir-1307_chr9_103084665-103084813_-

CAUCAAGACCCAGCUGAGUCACUGUCACUGCCUACCAAUCUCGACCGGACCUCGACCGGCUCGUCUGUGUUGCCAAUCGACUCGGCGUGGCGUCGGUCGUGGUAGAUAGGCGGUCAUGCAUACGAAUUUUCAGCUCUUGUUCUGGUGAC

(((((..((..(((((((((..(((.((((((((.(.(((.(((((((.((.((.(((..(((..((......))..)))..))))).))..))))))).))).).))))))))...)))...))...)))))))...))..))))).. (MFE:-57.30)

>rheMac2_mml-mir-4295_chr9_112294563-112294647_+

CUUUGUGGAACAGUGCAAUGUUUUCCUUGCCUGUGGCAAGACCACUUCAGUUCAAGGCUAAGAAACUAGACUGUUCCUACAGAGA

((((((((((((((.(...(((((....((((((((.....)))).........))))...)))))..))))))))).)))))). (MFE:-29.10)

>rheMac2_mml-mir-3663_chr9_116788609-116788705_-

CCCAUGACCUUAGUCCAGGCGCCUGCCCGAAUCGUGCUCGGGUGGAUAAGUCUGAUCUGAGCACCACAGGGGCCGGGCGCCUGGACCAAGGGGGUUC

(((....((((.((((((((((((((((.....(((((((((((((....))).)))))))))).....))).))))))))))))).)))))))... (MFE:-59.20)

>rheMac2_mml-mir-3941_chr9_122034284-122034385_+

GAGUCAGAAUUCUCAUCAGGCUGUGAUGCUCUGUUGUGUGUAGAUUGAAAGCCCUAAUUUACACACAACUGAACAUCAUAGCCUGAUGGUUCCUUUUUGUUU

....(((((...((((((((((((((((.((.(((((((((((((((.......))))))))))))))).)).)))))))))))))))).....)))))... (MFE:-49.50)

>rheMac2_mml-mir-548f-5_chrX_30557934-30558020_-

UAUUAGGUUGCUGCAAAAGUAAUUGUGGUUUUUUUCCAUUGAAAGUAAUGGGGAAAACCGUAAUUACUUUUGCACCAAACUCAUAGC

(((.((.(((.((((((((((((((((((((.(..((((((....))))))..))))))))))))))))))))).))).)).))).. (MFE:-34.80)

>rheMac2_mml-mir-3937_chrX_37404729-37404834_+

AGAAAAAUGCCCAGCCAGCCCUCAGUUGCUACAGCCCUCUAUUGUUUCAGCUCAACAACAACAGGGGGCUGUAGCAAUGGGGGGCUGGAUGGGCAUCUCAAUGUGC

.((...(((((((.(((((((((.((((((((((((((((.(((((..........))))).)))))))))))))))).))))))))).))))))).))....... (MFE:-65.70)

>rheMac2_mml-mir-500b_chrX_47647182-47647260_+

CCCCCUGUCUAAUCCUUGCUACCUGGGUGAGAGUGCUUUCUGAAUGCAGUGCACCCAGGCAAGGAUUCUGCAAGGGGGA

((((((((..(((((((((...(((((((..(.(((.........))).).))))))))))))))))..)).)))))). (MFE:-43.00)

>rheMac2_mml-mir-1468_chrX_62639446-62639531_-

GGCGGGCGGUUUCUCCGUUUGCCUGUUUUGCUGAUGUACAUUCAACUCAUUCUCAGCAAAAUAAGCAAAUGGAAAAUUUGUCCAUC

...((((((.((.(((((((((.(((((((((((..................))))))))))).))))))))).)).))))))... (MFE:-33.77)

>rheMac2_mml-mir-676_chrX_68990102-68990168_+

GCAUGACUCUUCAACCUCAGGACUUGCAGAAUUAAUGGAAUGCCGUCCUAAGGUUGUUGAGUUGUGC

(((..((((..((((((.(((((..(((............))).))))).))))))..))))..))) (MFE:-30.70)

>rheMac2_mml-mir-1912_chrX_113173443-113173524_+

CUCUAGGAUGUGCUCAUUGCAUGGGCUGUGUAUAUUAUUAUUCAAUACCCAGAGCGUGCAGUGUGAACAUUAAAUAGAGAUU

(((((.(((((.(.(((((((((..(((.((((...........)))).)))..))))))))).).)))))...)))))... (MFE:-29.90)

>rheMac2_mml-mir-1264_chrX_113174574-113174642_+

AGGUCCUCAAUAAGUAUUUGUUGAAAGAAUAAAUAAACCAACAAGUCUUAUUUGAGCACCUGUUAUGUG

((((.(((((((((.((((((((...............)))))))))))).))))).))))........ (MFE:-19.16)

>rheMac2_mml-mir-1298_chrX_113236403-113236514_+

AGACGAGGAGUUAAGAGUUCAUUCGGCUGUCCAGAUGUAUCCAAGUAUCCUCUGUUAUUUGGCAAUAAAUACAUCUGGGCAACUGACUGAACUUUUCGCUUUUCAUGACUCA

....(((((((.(((((((((.((((.((((((((((((((((((((........)))))))......))))))))))))).)))).))))))))).)))))))........ (MFE:-49.30)

>rheMac2_mml-mir-1911_chrX_113271153-113271232_+

UCGGCAUCUGCUGAGUACCGCCAUGUCUGUUGGGCAUCCACAGUCUCCCACCAGGCAUUGUGGUCUCUGCUGACGCUUUG

..(((.((.((.(((.(((((.(((((((.((((...(....)...)))).))))))).)))))))).)).)).)))... (MFE:-33.80)

>rheMac2_mml-mir-1277_chrX_116634664-116634741_+

ACCUCCCAAAUAUAUAUAUAUAUGUACGUAUGUGUAUAUAAAUGUAUACGUAGAUAUAUAUGUAUUUUUGGUGGGUUU

(((..(((((.((((((((((((.((((((((..(......)..)))))))).)))))))))))).)))))..))).. (MFE:-34.60)

>rheMac2_mml-mir-3672_chrX_119499314-119499397_+

UAUUUGUGAUUACCAUGAGACUCGUAUAAAACAUCUUAAAGACUAUUACAAGAUGUUUUACGAGUCUCAUGUUAAUCACAAAGA

..((((((((((.(((((((((((((..(((((((((............)))))))))))))))))))))).)))))))))).. (MFE:-40.60)

>rheMac2_mml-mir-320d-2_chrX_139189584-139189631_-

UUCUCUUCCCAGUUCUUCUUGGAGUCAAGAAAAGCUGGGUUGAGAGGC

(((((..(((((((.((((((....)))))).)))))))..))))).. (MFE:-23.40)

>rheMac2_mml-mir-891a_chrX_144153010-144153088_-

CCUUCAUCCUUGCAACGAACUUGAGCCAUUGAUUCAGUAAAACAUUCAAUGGCACAUGCUUGUUGUUAGGAUCAAAAGA

.(((.(((((.(((((((.(.((.((((((((.............)))))))).)).).))))))).)))))...))). (MFE:-27.02)

>rheMac2_mml-mir-514b_chrX_145369798-145369877_-

CAUGUGGUACUCUUCUCAAGAGGGAGGCAAUCAUGUGUAAUUAGAUAUGAUUGACACCUCUGUGAGUAGAGUAACACAUG

((((((.((((((.(((((((((....(((((((((.(....).)))))))))...))))).)))).)))))).)))))) (MFE:-38.00)

>rheMac2_mml-mir-2114_chrX_148334327-148334406_+

CCUUCAUGCUCCUAGUCCCUUCCUUGAAGCGGUCGGAUAAUCACAUGACGAGCCUCAAGCAAGGGACUUCAAGCUGGUGG

((.(((.(((...((((((((.(((((.((.((((..........))))..)).))))).))))))))...)))))).)) (MFE:-31.50)

>rheMac2_mml-mir-3202-1_chrX_152105076-152105156_+

UAUUAAUAUGGAAGGGAGAAGAGCUUUAAUGAUUGGAGUCAUUUUCAGAGCAUUAAAGCUGUUCUCCCUUCCAUAUUAAUG

((((((((((((((((((((.((((((((((.((((((....))))))..)))))))))).)))))))))))))))))))) (MFE:-47.80)

>rheMac2_mml-mir-3202-2_chrX_152105077-152105155_-

AUUAAUAUGGAAGGGAGAACAGCUUUAAUGCUCUGAAAAUGACUCCAAUCAUUAAAGCUCUUCUCCCUUCCAUAUUAAU

(((((((((((((((((((.((((((((((....((.......))....)))))))))).))))))))))))))))))) (MFE:-40.20)

>rheMac2_mml-mir-3616_chr10_17303653-17303744_-

UAUCGCUCUGCUGGCAUCAUGAAGUGCACUCAUGAUAUGUUUGCCCCAUCAGGCUAUCAUGAGUGCACUUCACAAUGCAGGCGGAGUUGGCA

....((((((((.((((..((((((((((((((((((.(((((......)))))))))))))))))))))))..)))).))))))))..... (MFE:-52.50)

>rheMac2_mml-mir-3617_chr10_18776212-18776290_+

AGGUCAUAGAAAGACAUAGUUGCAAGAUGGGAUUAGAAACCAUAUGUCUCAUCAGCACCCUAUGUUCUUUCUGUGCCCU

(((.(((((((((((((((.(((..((((((((............)))))))).)))..))))).)))))))))).))) (MFE:-32.00)

>rheMac2_mml-mir-3646_chr10_20042178-20042261_-

UUCAGUGGGCUGGGUUCAUUUCAUCUUCAUGACAACCCUGUGUGGGAAAAUGUCAUGAAAAUGAAAGGAGACCAGCCCAUUGAA

.((((((((((((.(((.((((((.(((((((((.(((.....)))....))))))))).)))))).))).)))))))))))). (MFE:-47.40)

>rheMac2_mml-mir-3193_chr10_32963177-32963228_-

UCCUGGGUAGGAUCUGAGGAGUGGAAUCUCAUUCCCCAGCUCCUGAGCAGGA

(((((..(((((.(((.(((((((....))))))).))).)))))..))))) (MFE:-26.70)

>rheMac2_mml-mir-3192_chr10_51801585-51801660_+

GGAAGGGAUUCUGGGAGGUUGUAGCAGUGGAAAAAUUCCUUUUUUCCUCUGAUCGCCCUUUCAGCUCUUUCCUUCU

((((((((..(((..(((.((...(((.(((((((.....))))))).)))..)).)))..)))....)))))))) (MFE:-32.30)

>rheMac2_mml-mir-3118-5_chr10_59018434-59018512_-

CACACACACUACAAUAAUUUUCAUAAUGCCAUCACGCACAAUCACCAUAUGACUGCAUUAUGAAAAUUAUUGUAGUAUU

.......((((((((((((((((((((((..(((...............)))..))))))))))))))))))))))... (MFE:-28.66)

>rheMac2_mml-mir-3118-1_chr10_59018434-59018512_-

CACACUACAAUAAUUUUCAUAAUGCCAUCACGCACAAUCACCAUAUGACUGCAUUAUGAAAAUUAUUGUAGUAUU

...((((((((((((((((((((((..(((...............)))..))))))))))))))))))))))... (MFE:-28.66)

>rheMac2_mml-mir-3118-2_chr10_59018435-59018507_-

ACACUACAAUAAUUUUCAUAAUGCCAUCACGCACAAUCACCAUAUGACUGCAUUAUGAAAAUUAUUGUAGUAU

..((((((((((((((((((((((..(((...............)))..)))))))))))))))))))))).. (MFE:-28.66)

>rheMac2_mml-mir-3618_chr10_64151601-64151688_+

UAAGCUGAGUGCAUUGUGAUUUCCAAUAAUUGAGGCAGUGGUUCUAAAAGCUGUCUACAUUAAUGAAAAGAGCAAUGUGGCCAGCUUG

(((((((..((((((((..(((.((.((((..(((((((..........)))))))..)))).)).)))..))))))))..))))))) (MFE:-29.20)

>rheMac2_mml-mir-548j_chr10_70393738-70393850_-

GGGAAGCCAGUAAACAGUUAGGCUGGUGCAAAUGUAAUUACGGUCUUUGAUAUUACUUUCAGUGGCAAAAACUGCAUUACUUUUGCACCAGCCUACUAGAACACUGAGCUUAG

...((((((((....(((.(((((((((((((.(((((..((((.((((.((((......)))).)))).)))).))))).)))))))))))))))).....)))).)))).. (MFE:-44.10)

>rheMac2_mml-mir-3199-1_chr10_71750678-71750766_-

GGUGACUCCCUGGAACUGCCUUAGGAGAAAGUUUCUGGAAGUUUUGACAUUCCAGAAACCUUCUCCUAAGGCAGUCCCUGGGAGUCACU

((((((((((.((.(((((((((((((((.((((((((((((....)).)))))))))).))))))))))))))).)).)))))))))) (MFE:-65.50)

>rheMac2_mml-mir-3199-2_chr10_71750679-71750765_+

GUGACUCCCAGGGACUGCCUUAGGAGAAGGUUUCUGGAAUGUCAAAACUUCCAGAAACUUUCUCCUAAGGCAGUUCCAGGGAGUCAC

(((((((((.(((((((((((((((((((((((((((((.((....))))))))))))))))))))))))))))))).))))))))) (MFE:-70.60)

>rheMac2_mml-mir-3653_chr10_73160089-73160198_-

UCCCUGGGGACCCCUGGCAGCCCCUCCUGAUGAUUCUUCUUCCUGAGCACGCUCAUGAUGAGCAAACUGAGCCUCUAAGAAGUUGACUGAAGGGGCUGCUUCCCCAGGGA

((((((((((.....((((((((((.(........((((((...(((...(((((............)))))))).))))))......).)))))))))))))))))))) (MFE:-52.14)

>rheMac2_mml-mir-3200_chr10_74603916-74604000_+

GGUGGUCGAGGGAAUCUGAGAAGGCGCACAAGGUUUGUGUCCAAUACAGUCCACACCUUGCGCUACUCAGGUCUGCUCGUGCCCU

((.(((((((.(.(((((((..((((((..((((..(((..(......)..))))))))))))).))))))).).)))).))))) (MFE:-34.50)

>rheMac2_mml-mir-3909_chr10_79247253-79247371_+

GGUUUGCUGUUGCACCAUCAUUCCUCUGGGGAGCAGGCCCUGGGAGACAGGGAAAAGCACACCAGGAGCUUGUCCUCUAGGGCCUGCAGUCUCACAGGAGUGUGACGUGCACCGAGACC

(((((.(.((.((((...(((((((.(((((.(((((((((((..((((((.................))))))..)))))))))))..))))).)))))))....)))))).)))))) (MFE:-58.13)

>rheMac2_mml-mir-3201_chr10_92296082-92296134_+

GGAAUGUGAAGAAAAAUAAGAGGCUAGGAUUAGCCUCUUAUUUUUACAUGCCU

((.(((((((....((((((((((((....))))))))))))))))))).)). (MFE:-22.50)

>rheMac2_mml-mir-1302-10_chr11_10197-10333_+

GGAUGCCCAGUUAGUUUGAAUUUUAGAUAAACAACGAAGAAUUUCUUAGCAUAAAUAUGUCCCAAGCUUAGUUUGGGACAUACUUAUGCUAAAAGUAUUAUUGGUGGUUUAUCUGAGAUUCAAAAUUAAGCAUUUUA

((((((....((((((((((((((((((((((.((.((.(((...(((((((((.((((((((((((...)))))))))))).)))))))))....))).)).)).))))))))))))))).))))))))))))).. (MFE:-55.10)

>rheMac2_mml-mir-1291_chr11_45776505-45776591_-

GGUAGAAUUCCAGUGGCCCUGACUGAAAACCAGCAGUUGUACUGUAGCUGUUGGUUUCAAGCAGAGGCCUAAAGGACUGUCUUCCUA

((.(((..(((...(((((((.((..(((((((((((((.....)))))))))))))..))))).))))....)))...))).)).. (MFE:-38.70)

>rheMac2_mml-mir-3198_chr11_51333723-51333802_-

GAAUCUGUUCUCACUGUUCACCCAGCACUAGCAGUACCAGACGGUUCUGUGGAGUCCUGGGGAAUGGAGAGAGCACAGUC

.....(((((((.((((((.(((((.(((.((((.(((....))).))))..))).))))))))))).)))))))..... (MFE:-36.90)

>rheMac2_mml-mir-1228_chr11_54262407-54262479_+

GUGGGCAGGGGCAGGUGUGUGGUGGGCGGUGGCCUGCGGUGAGCACGGCCUUCACACCUGCCUCGCCCCCCAG

..((((.((((((((((((....((((.((((((...)))...))).)))).))))))))))))))))..... (MFE:-44.10)

>rheMac2_mml-mir-548cb_chr11_61637521-61637617_+

CAUUGGCAUCUAUUAGGUUGGUACAAAACUAACUGCAGUUUUUGCCAUUACUUUCAGUAGCAAAAAUCUCAAUUACUUUUGUACCAACUUAAUACUU

..........((((((((((((((((((.(((.((..((((((((...(((.....)))))))))))..)).))).))))))))))))))))))... (MFE:-32.20)

>rheMac2_mml-mir-3913-1_chr11_66573277-66573378_-

UUGUUUAUAAUAAACUGAAAUAUUUGGGACUGAUCUUGACACUCUUACAUGUUUUGGCAGACAUCAAGAUCAGUCCCAAAUAUUUCAGUUUAUUAUAGACAG

.((((((((((((((((((((((((((((((((((((((...(((..((.....))..)))..)))))))))))))))))))))))))))))))))))))). (MFE:-59.60)

>rheMac2_mml-mir-3913-2_chr11_66573278-66573377_+

UGUCUAUAAUAAACUGAAAUAUUUGGGACUGAUCUUGAUGUCUGCCAAAACAUGUAAGAGUGUCAAGAUCAGUCCCAAAUAUUUCAGUUUAUUAUAAACA

.((.(((((((((((((((((((((((((((((((((((((((..((.....))..))).)))))))))))))))))))))))))))))))))))).)). (MFE:-56.90)

>rheMac2_mml-mir-1252_chr11_76445916-76445980_+

AGAAAGAAGGAAGCUGAAUUCAUUUAGAAAAGAGAAUGCCAAAUGAGCUUAAUUUCCUUUUUUCU

(((((((((((((.(((.(((((((.(...........).))))))).))).))))))))))))) (MFE:-18.40)

>rheMac2_mml-mir-617_chr11_77769934-77770016_-

CAUCAUAAGGAGCCUGGACUUACCAUAUGAAGGUGGUAGGAAAUGGGAAGUCUAGGCUCCUUCUGAUUCAAUAAAUGAGGAAC

.((((.(((((((((((((((.((((................)))).))))))))))))))).))))................ (MFE:-32.99)

>rheMac2_mml-mir-3922_chr11_105706113-105706196_+

GGAAGAGUCAAGUCAAGGCCACAGGUCCUACAACAGGGCUGGAAAGCACACCUGUGGGACCUCUGGCCUUGGCUUGACUCUUUC

(((((((((((((((((((((.((((((((((.....(((....))).....)))))))))).))))))))))))))))))))) (MFE:-60.90)

>rheMac2_mml-mir-4304_chr11_124278904-124278965_-

AGAGAAAUGGCUAGCAUGUCCAGGGCAUCCCCAUUGCUCUGUGACCACUGCCAUCCUUCUCC

.((((((((((.((...((((((((((.......))))))).)))..)))))))..))))). (MFE:-25.60)

>rheMac2_mml-mir-3612_chr11_129566966-129567052_+

GAGACUGGGGAUGAGGAGGCACCUUGAGAAGUGGAAGGAAUGGGAUCUACUUCCAGUUCACUAGAGGCAUCUCGACACCCCCAGCUC

(((.((((((.((..((((..((((..(((.((((((............)))))).)))....))))..))))..)).))))))))) (MFE:-36.80)

>rheMac2_mml-mir-3128_chr12_40875220-40875283_-

UUCCUCUUGCAAGUAAAAAACUCAUUUUUUAAGGAAAAUGAGAGUUUUUUACUUGCAGUAGGAA

.((((.(((((((((((((((((((((((....))))))..))))))))))))))))).)))). (MFE:-29.30)

>rheMac2_mml-mir-1258_chr12_43490621-43490693_-

CUCUGGCUUCCACGACCUAAUCCUAACUCCUGCGAGUCCCUGGAGUUAGGAUUAGGUGGUGGAAGCCACAGGA

((.((((((((((.((((((((((((((((...........)))))))))))))))).)))))))))).)).. (MFE:-50.80)

>rheMac2_mml-mir-1245_chr12_52619651-52619720_+

AUUUAUGUAUAGGCCUUUAGCUCAUCUAAUGUUGAAUACUCUUUAGAUGAUCUAAAGGCCUACACAUAAA

.(((((((.(((((((((((.((((((((.((.....))...)))))))).))))))))))).))))))) (MFE:-33.90)

>rheMac2_mml-mir-2355_chr12_71005065-71005151_-

CAGACGUGUCAUCCCCAGAUACAGUGGACAAUAUGCUAUUAUAAUUGUAUGGCAUUGUCCUUGCUGUUUGGAGAUAAUACUGCUGAC

(((..((((.(((.((((((((((.(((((..(((((((.........))))))))))))))).))))))).))).))))..))).. (MFE:-31.40)

>rheMac2_mml-mir-1302-4_chr12_71164776-71164925_-

AAUGCAGAAGCACAGCUUAAAUUUGAAUUUCAGAUAAACAAAUUUUUCUUAGAAUAAGUAUGUCCCCAUGCAACAUUUGGGACAUACUUAUGCUAAAACAUUAUUUGUGUUUCAUCUGAAAUUCAAAUUCAACUGGACAUCCUGUAUUUU

(((((((..(..(((.((.((((((((((((((((..((((((.....((((.(((((((((((((.(((...)))..))))))))))))).))))......)))))).....)))))))))))))))).)))))..)...))))))).. (MFE:-52.20)

>rheMac2_mml-mir-548f-2_chr12_76199544-76199641_-

UAAUAACUAUUAAGUUGGUGCAAACAUAAUUGUGGUUUUUGUCAUUACUUUUAAUGGCAAAAACUGUAAUUACGUUUGCACCAACCUAAUAUUUUAAU

.......(((((.((((((((((((.((((((..(((((((((((((....)))))))))))))..)))))).)))))))))))).)))))....... (MFE:-45.10)

>rheMac2_mml-mir-3132_chr12_83397139-83397213_-

GGUGGGAUGGGUAGAGAAGGAGCUCAGAGGACUGUGCGCCUUAUUUCCCCUGAGCCCUCCCUCUCUCUUCCCACC

(((((((.(((.((((.(((.((((((.(((..(((.....))).))).)))))))))..))))))).))))))) (MFE:-38.40)

>rheMac2_mml-mir-3133_chr12_105406245-105406319_+

CAGAAUUUGCUAGGAACUCUUAAAACCCAAAAGUAAAAGACAACCUGUUGAGUUUUAAGGGUUCUUUAUAUUCUG

((((((.....(((((((((((((((.(((..((........))...))).)))))))))))))))...)))))) (MFE:-24.20)

>rheMac2_mml-mir-548s_chr13_11834250-11834331_+

UUACUACAGAAAUAACUACAGUUUUUGCCACUAUUUUUAAUAAUUAUAAUAAUGGCCAAAACCGCAAUUAUUUUUGCACCAA

......(((((((((...(.(((((.((((.((((............)))).)))).))))).)...)))))))))...... (MFE:-15.20)

>rheMac2_mml-mir-3681_chr13_12262729-12262800_+

UCUUCCAGUAGUGGAUGAGGCACAGUGUGCAGGGUCAACUGUGCAUGCAGUGCUUCAUCCACUACCAGAAGU

.((((..((((((((((((((((.(((((((.((....)).))))))).))))))))))))))))..)))). (MFE:-45.50)

>rheMac2_mml-mir-3682_chr13_54127689-54127771_-

UAAGUUAUAUAUGUCUACCUCUACCUGCGUUAACAUAACAUAAUAAAGGUGUCAUGGUACAGGAGGUAGACAUAUAUAACUUA

((((((((((((((((((((...((((......(((.((((.......)))).)))...)))))))))))))))))))))))) (MFE:-34.70)

>rheMac2_mml-mir-217_chr13_56295868-56295977_-

AAUAUAAUUAUUACAUAGUUUUUGAUGUCGCAGAUACUGCAUCAGGAACUGAUUGGAUAAGAAUCAGUCACCAUCAGUUCCUAAUGCAUUGCCUUCAGCAUCUAAACAAG

.................((((..(((((.(.((.((.(((((.((((((((((.((((........))).).)))))))))).))))).)).)).).))))).))))... (MFE:-32.80)

>rheMac2_mml-mir-3126_chr13_69341085-69341158_+

AUGAUUAUAUGAGGGACAGAUGCCAGAAGCACUGGUUAUGAUUUGGAUCUGGCAUCCCUCACACAGAUAAUUAU

((((((((.(((((((....(((((((....(..(.......)..).)))))))))))))).....)))))))) (MFE:-24.10)

>rheMac2_mml-mir-3127_chr13_97220531-97220603_+

GGCCAGGCCCAUCAGGGCUUGUGGAAUGGGAAGAAGGGAUCCUUCCCCUUCUGCAGGUCUGCUGGGUGCGGCU

((((..(((((...(((((((..(((.((((((........)))))).)))..)))))))..)))))..)))) (MFE:-40.60)

>rheMac2_mml-mir-4265_chr13_108948198-108948296_-

UGCAGUGGGUUGGAGCUUCAGCCUACACCUGUAAAGAACUGGUCAGCCUGGGGACUGGUGAUCUCUGCAGCUGUGGGCUCAGCUCUGGGCUGGACCUGG

..((((((.(..(((((..((((((((.(((((.(((((..(((........)))..))..))).))))).)))))))).)))))..).)))...))). (MFE:-43.50)

>rheMac2_mml-mir-3679_chr13_118038170-118038236_-

CCCGGUGAGGAUAUGGCAGGGAAGAGGAGUUUCCCUCUGUCCCCUUCCCCCAGUAAUCUUCAUCAUG

...(((((((((.(((.((((.(((((......)))))...))))....)))...)))))))))... (MFE:-26.40)

>rheMac2_mml-mir-3664_chr14_3894773-3894869_+

CUGUAAACUUGAAGGCAGGGAACUCUGUCUUCACUCGAGUGCCCUCCAACAUGAGCACUCAGGAGUGGUGACAGAGUUCCCUCCCUUCAACGUGGAU

......(((((((((.(((((((((((((.((((((((((((.(........).))))))..)))))).))))))))))))).))))))).)).... (MFE:-53.80)

>rheMac2_mml-mir-548k_chr14_4489614-4489728_-

CUUUUCUCAAGUAUUGCUAUUAGGUUCGUGCAAUAGUGUUUGUGUUUUUGCUUUAUUUUUAAUGGCAAAAACCGCAAUUAUUUUUCCUUCCACCUAAUAUGAUGCAAAGUUGGCU

..........((((((.((((((((.........((((.((((((((((((((((....))).)))))))).))))).)))).........)))))))))))))).......... (MFE:-25.77)

>rheMac2_mml-mir-3164_chr14_5677446-5677528_-

CUUGGAAACUGUGACUUUAAGGGAAAUGACGCACAGCAGGCCCUGGAAUCACGCCGUUUUGCUUGAAGUUGCAGUUUCCCAGG

((.((((((((..((((((((.((((((.((..(((......)))......)).)))))).))))))))..)))))))).)). (MFE:-35.80)

>rheMac2_mml-mir-3163_chr14_7515721-7515793_+

UUCCUCAUGUGUAAAAUGAGGGCAUUAAGACCUUCCUUCCUUGUAUUACUGCCCCCAUUUUAUAGAUGAGGAA

.(((((((.(((((((((.(((((.(((.((...........)).))).))))).))))))))).))))))). (MFE:-30.90)

>rheMac2_mml-mir-3159_chr14_53500744-53500817_-

CCAAAGUCCUAGGAUUAUAAGUGUCAGCCACGGGCCGGGCACAGUGGCUCACUCCUGUAAUCCCAGCACUUUGG

(((((((.((.((((((((.(.((.((((((..((...))...)))))).)).).)))))))).)).))))))) (MFE:-33.20)

>rheMac2_mml-mir-3165_chr14_70113039-70113113_-

CCGGUGGCAAGGUGGAUGCAAUGUGACCUCAACACUUGGUCCUCUGAGGUCACAUUGUAUCCACCUUACCACUGG

(((((((.(((((((((((((((((((((((.............))))))))))))))))))))))).))))))) (MFE:-52.92)

>rheMac2_mml-mir-1304_chr14_92296013-92296103_-

AAUCCCUUGAGCCCAGUGGUUCGAGGCUACAAUGAGAUGUGAUCCUGCCACAUCUCACUGUAGCCUCAAACCCUUGGGCUCAAGCGAUUCA

((((.(((((((((((.((((.(((((((((.(((((((((.......))))))))).))))))))).)))).))))))))))).)))).. (MFE:-59.30)

>rheMac2_mml-mir-548l_chr14_93015670-93015755_-

UAUUAGGUUGGUGCAAAAGUAUUUGCGGGUUUUGUCAUUGAAAGUAAUGGCAAAAACUGCAAUUACUUCUGCACCAACCUAAUGCU

(((((((((((((((.(((((.((((((.(((((((((((....))))))))))).)))))).))))).))))))))))))))).. (MFE:-49.00)

>rheMac2_mml-mir-1260b_chr14_94898488-94898576_+

UCUCCGUUUAUCCCACCACUGCCACCAUUAUUGCUACUGUUCAGCAGGUGCUGCUGGUGGUGAUGGUGAUAGUCUGGUGGGGGCGGUGG

.(.((((...(((((((((((.(((((((((..(((.....((((....)))).)))..))))))))).))))..))))))))))).). (MFE:-42.90)

>rheMac2_mml-mir-3920_chr14_100126450-100126535_-

ACUGAGUGAAGGGGUCAGAGAGUUAAGAGAAUUAGUACAGGUGAGAUUGUACUGAUUCUUUUAACUCUCUGACCGCCUCGCUCAGU

(((((((((.((((((((((((((((((((((((((((((......)))))))))))))))))))))))))))).))))))))))) (MFE:-60.90)

>rheMac2_mml-mir-3167_chr14_125129595-125129679_-

GGCUAUUGAGGCACCAGUAUUUCUGAAAUUCUAUUUUCUGAAGUUCUUCAGGAAGGAUUUCAGAAAUACUGGUGCCUGGACAGCU

((((.((.((((((((((((((((((((((((...(((((((....))))))))))))))))))))))))))))))).)).)))) (MFE:-52.60)

>rheMac2_mml-mir-3621_chr15_1075500-1075584_+

GUGAGCUGUUAGGGAUGCGGGUUGGGGUCUGCAGGGCGGUGCGGCCGCUGCCGCCUGAUGCCACGCCCUUGUCUGUGUCCCACAG

.....((((..((((((((((..(((((..((((((((((((....)).)))))))..)))...)))))..)))))))))))))) (MFE:-43.90)

>rheMac2_mml-mir-3154_chr15_10635149-10635232_+

GGCCCCUCCUUCUCAGCCCCAGCUCCCGCUCACCCCUGCCACGUCAAAGGAGGCAGAAGGGGAGUUGGGAGCAGAGAGGGGACC

((((((((..(((..((((((((((((.((.....(((((.(......)..))))).)))))))))))).))))))))))).)) (MFE:-44.80)

>rheMac2_mml-mir-3927_chr15_26688517-26688587_+

UGCCAAUGCCUAUCAAAUAUCUACCUGUCCUAUGACAAACAUGACAGGUAGAUAUUUGAUAGGCAUUGGCA

.((((((((((((((((((((((((((((..(((.....))))))))))))))))))))))))))))))). (MFE:-51.10)

>rheMac2_mml-mir-1302-8_chr15_38854157-38854285_+

CCCAUUUAAGUUUGAAUUUCAUAUAAACAGAGUAAUUUUCAGUAUUAGUGUAUCACGUGCAGCAUUUGGGACAUACUUAUGCUAAAAAAAUUAGGUGGUGUUGAUCUGAAAUUCCAGUGUAGAUGGGCA

((((((((..((.((((((((.((.(((((..(((((((.(((((.(((((.(((.(((...))).)))...))))).)))))...)))))))..)..)))).)).)))))))).))..)))))))).. (MFE:-31.20)

>rheMac2_mml-mir-873_chr15_48227767-48227843_+

GUGUGCAUUUGCAGGAACUUGUGAGUCUCCUAUUGAAAAUGAACAGGAGACUGAUGAGUUCCCGGGAACACCCACAA

(((.(..(((.(.((((((..(.((((((((.((.......)).)))))))).)..)))))).).)))..).))).. (MFE:-31.10)

>rheMac2_mml-mir-3152_chr15_58388681-58388753_-

GUGCAGAGUUAUUGUCUCUGUUCUAACACAAGACGAGGCUUCCCUGUGUUAGAAUACGGCAAUAACUCUGCAC

((((((((((((((((..((((((((((((.((.......))..)))))))))))).)))))))))))))))) (MFE:-39.00)

>rheMac2_mml-mir-3153_chr15_97818571-97818645_+

GACAGAUUUUAAAUGUCCCUAUCCCCCAAUUAAACUAGACUGGGGGAAAGCGGGUAGGGACAUUUAAAAUUUGUU

((((((((((((((((((((((((((((.((......)).))))))).......))))))))))))))))))))) (MFE:-38.51)

>rheMac2_mml-mir-4290_chr15_101881083-101881178_-

GUCACCAAGAAGGUGCGAGGGAGGGGGCAGUCCCGAUCUGAAUCCCACCAAAAUAGGUGGUAGAGGGCUGCCUCCUUCCUUCCCUCACCUCUGACC

((((....((.((((.(((((((((((((((((...((((....(((((......))))))))))))))))))))))))))....)))))))))). (MFE:-49.10)

>rheMac2_mml-mir-3651_chr15_104196510-104196599_-

GAUUCGAUGGGCCAUAGCAAUCCUGUGAUUUAUGCAUGGAGGCUGCUUCUCCUCAGCAGCCGCCAUAGCCCGGUCACUGGUACAUGGUUC

........(((((((......((.((((((...((((((.(((((((.......))))))).)))).))..)))))).))...))))))) (MFE:-34.50)

>rheMac2_mml-mir-1288_chr16_16183356-16183430_-

GAGGGUGUUGAUCAGCAGAUCAGGACUGUAACUCACCAUAGUGGUGGACUGCCCUGAUCUGGAGACCACUGCCUU

.(((((..((.((..(((((((((...(((..(((((.....)))))..))))))))))))..)).))..))))) (MFE:-29.60)

>rheMac2_mml-mir-1180_chr16_18891348-18891416_-

GCUGCCGGACCCACCCGGCCGGGAAUAGUGCUCCUGGUUGUUUCCGGCUCGCGUGGGUGUGUCGGCGGC

((((((((((((((.((((((((((((..((.....)))))))))))).)).))))))...)))))))) (MFE:-41.20)

>rheMac2_mml-mir-3185_chr16_32949153-32949217_-

GAAUGGAAGAAGGCGGUCGGGCUCCGGGAGCCAGGCCGCAGCGCCAUCCGCCUUCUGUCCAUGUC

..((((((((((((((..(((((.(((........))).))).))..))))))))).)))))... (MFE:-35.10)

>rheMac2_mml-mir-3614_chr16_41110454-41110539_-

GGUUCUGUCUUGGGUCACUUGGAUCUGAAGGCUGCCCCUUUGCUCUCUGGGGUAGCCUUCAGAUCUUGGUGUUUUGAUUACUCACU

(((...(((......((((.((((((((((((((((((..........)))))))))))))))))).))))....))).))).... (MFE:-42.90)

>rheMac2_mml-mir-2117_chr16_53463099-53463178_+

GUUCUGAUCUGCAUCUGUCCGACAUGGUAAACAGCAGGAAUGACUGUAGCUGUUCUCUUUGCCAAGGACAGAUCUGAUCU

.....((((.(.((((((((....(((((((((((((......))...))))).....)))))).))))))))).)))). (MFE:-28.00)

>rheMac2_mml-mir-1250_chr16_76583141-76583252_-

UUGUCUCGCUGGCCUGGAAGGUGACGGUGCUGAAUGUGGCCUUUUUGCCUUUUCCAAAGGCCACAUUUUCCAGCCCAUUCACCCUUCCAGAGCCCUCUGAGGUGGCCUAGGC

(..(((((..((((((((((((((.((.((((((((((((((((...........))))))))))))...))))))..))).)))))))).)))...)))))..)....... (MFE:-51.70)

>rheMac2_mml-mir-3186_chr16_76909157-76909241_-

AGCCCGCGGUUCCCUCAGACGUCUGUCCAUGUGGCUUUAACCAAGUUCAAAGUCACGUGGAGAGAUGGCUUUGGAACUGUGGGCU

(((((((((((((...((.(((((.((((((((((((((((...))).))))))))))))).))))).))..))))))))))))) (MFE:-51.90)

>rheMac2_mml-mir-4305_chr17_18734520-18734617_-

CUGCCUUAGACCUAGACACCUCCAGUUCUGGGUUUUUAGAGGCCUAAUCCUCUACAAACUCAGUUUUCAGACUGUGAGGAAAAUUCUCUUAUUGCUUU

..((...(((........(((((((((((((((((.((((((......)))))).))))))))......))))).)))).......)))....))... (MFE:-28.76)

>rheMac2_mml-mir-3613_chr17_28813561-28813647_-

UGGUUGGGUUUGGAUUGUUGUACUUUUUUUUUUGUUCGUUGCAUUUUUAGGAACAAAAAAAAAAGCCCAACCCUUCACACCACUUCA

.((...(((.((((..((((..(((((((((((((((.(.........).)))))))))))))))..))))..)))).)))...)). (MFE:-27.60)

>rheMac2_mml-mir-3169_chr17_40319706-40319788_-

AUGUAAAAACAUAGGAUUAUGCUUGGCACAUAGUACAAAGUCUCAUGGUACUGUGUGCCAAGCAUAGUCCUGUGUUCUUACAU

((((((.(((((((((((((((((((((((((((((...........))))))))))))))))))))))))))))).)))))) (MFE:-53.90)

>rheMac2_mml-mir-1267_chr17_87725419-87725497_-

CUCACAAAUCUCCUGUUGAAAUGUGAUCCCCACCUCCAGCAUUGGGGUAUUACAUUUUAACAUGAGAUUUGGAUGAGGA

((((((((((((.(((((((((((((((((((..........))))).)))))))))))))).))))))))..)))).. (MFE:-37.80)

>rheMac2_mml-mir-4317_chr18_7925353-7925417_+

AAAAGGCGAGACAUUGCCAGGGAGUUUAUUUUGUAGCUCUCUUGAUAAAAUGUUUUAGCAAACAC

.....((((((((((.(.((((((((........)))))))).)....)))))))).))...... (MFE:-17.80)

>rheMac2_mml-mir-133a-1_chr18_14832030-14832117_-

ACAAUGCUUUGCUAGAGCUGGUAAAAUGGAACCAAAUCGCCUCUUCAAUGGAUUUGGUCCCCUUCAACCAGCUGUAGCUAUGCAUUGA

.((((((...((((.(((((((..((.((.((((((((............)))))))).)).))..))))))).))))...)))))). (MFE:-40.50)

>rheMac2_mml-mir-4320_chr18_43196198-43196260_-

GACGUGGGGUUUGCUGUAGACAUUUCAGACAACUCGGGAUUCUGCAGCUUCCUGGCAACUUUG

...((.(((...((((((((.((..(((....)).)..))))))))))..))).))....... (MFE:-22.60)

>rheMac2_mml-mir-3187_chr19_549406-549475_+

GCUGGCCCUGGGCAGCGUGUGGCUGAAGGUCACCAUGUUCUCCUUGGCCAUGGGGCUGCGUGGGGCCAGC

((((((((((.(((((..((((((.((((............))))))))))...))))).)))))))))) (MFE:-42.30)

>rheMac2_mml-mir-3940_chr19_6320054-6320155_-

GCUUAUCGAGGAGAAGAUCGAGGUGGGUUGGGGCGGGCUCUGGGGGUUUGGGCUCGCAGCCCAGAUCCCAGCCCACUCACCUCGGCUACUCUCCUUCCUUCC

.......(((((((((.(((((((((((..((((........(((((((((((.....))))))))))).)))))))))))))))))..)))))))...... (MFE:-55.40)

>rheMac2_mml-mir-3189_chr19_17992287-17992359_+

GCCUCAGUUGCCCCGUCUGUGCCCUGGGUGGGAACGUCCUGGACCCCCUUGGGUCUGAUGGGGUAGCUGAUGC

((.((((((((((((((...((((.(((.(((..(.....)..)))))).))))..)))))))))))))).)) (MFE:-43.80)

>rheMac2_mml-mir-1270-1_chr19_19969389-19969471_-

CAUAAUGUUAUACUGGAGAGCAGGAAGAGCUGUGUUGGGUAUAAGUAACAGGCUUUUCUUUAUCUUCUAUGUGACUCUUUGCA

......((((((..(((((..((((((((((.(((((........)))))))))))))))..)))))..))))))........ (MFE:-25.20)

>rheMac2_mml-mir-320e_chr19_53056120-53056172_-

GCUUUCUCUUCCCAGUUCUUCCUGGAGUCGGGGAAAAGCUGGGUUGAGAGGGU

((((((((..(((((((.(((((.(...).))))).)))))))..)))))))) (MFE:-27.70)

>rheMac2_mml-mir-512-1_chr19_59770293-59770376_+

UCUCACUCUGUGGCACUCAGCCUCGGGGGCACUUUCUGGUGUCAGAAUGAAAGUGCCGUCAUUGCUGAGAUCCAAUGACUGAGG

.((((.((..(((..((((((...((.(((((((((............))))))))).))...))))))..)))..)).)))). (MFE:-35.60)

>rheMac2_mml-mir-1323_chr19_59774855-59774927_+

ACUGAUGUCCUCAAAACUGAGGGGCAUUUUCUGUGAUUUGAAAGGAAAGUGCACCCAGUUUUGGGGAUGUCAA

..(((((((((((((((((.((.(((((((((...........))))))))).))))))))))))))))))). (MFE:-38.30)

>rheMac2_mml-mir-515-1_chr19_59779857-59779943_+

UCUCAUGCAGUCAUUCUCCAAUAGGAAGCACCAUCUGUGGUCUGAAAGCAAGCAGAGGGCCUCCUUUUGGAGCGUUACUGUUUGAGA

(((((.(((((.((.((((((.((((.((.((.(((((.((......))..))))))))).)))).)))))).)).))))).))))) (MFE:-38.70)

>rheMac2_mml-mir-519e_chr19_59782036-59782122_+

UCUCAUGCAGUCAUUCUCCAAUGGGAAGCACCUUCUGUGGUCUGAAAGCAAGCAGAGGGCCUCCUUUUGGAGCAUUACUGUUUGAGA

(((((.(((((.((.((((((.((((.((.(((.((((.((......))..))))))))).)))).)))))).)).))))).))))) (MFE:-38.20)

>rheMac2_mml-mir-515-2_chr19_59783542-59783628_+

UCUCAUGCAGUCAUUCUCCAAUGGGAAGCACCUUCUGUGGUCUGAAAGCAAGCAGAGGGCCUCCUUUUGGAGCAUUACUGUUUGAGA

(((((.(((((.((.((((((.((((.((.(((.((((.((......))..))))))))).)))).)))))).)).))))).))))) (MFE:-38.20)

>rheMac2_mml-mir-1283-1_chr19_59788215-59788300_+

CCCAGGCUGUGACUCUACAAAGGAAAGCACUUUCUGUUGUCGAAAGAAAAGAAAGCGCUUCCCUUUUGAGAAUUACGGUUUGAGAA

..((((((((((.(((..(((((.((((.((((((....((....))..)))))).)))).)))))..))).)))))))))).... (MFE:-36.30)

>rheMac2_mml-mir-519bb_chr19_59795287-59795367_+

CAGGCUCUAACCCUCUAGAGGGAAGCGCUUUCUGUCGUCUGAGAGAGAAGAAAGUGCAUCCUUUUAGAGGAUUACGGUUUG

((((((.(((.((((((((((((.(((((((((.((........))..))))))))).)))))))))))).))).)))))) (MFE:-40.00)

>rheMac2_mml-mir-526a-1_chr19_59830587-59830671_+

CUCAUGAUGUGACUCUCUAGAGGAAAGCGCUUUCUGUUGGCCAAAAGAAUAGGAAGCACUUCCCUUUAGAGUGUUACGCUUUGAG

((((...((((((.(((((((((.(((.((((((((((.........)))))))))).))).))))))))).))))))...)))) (MFE:-38.70)

>rheMac2_mml-mir-518a-1_chr19_59836260-59836346_+

UCUCAUGCUGUGACCCUCCAAAGGGAAACACUUUCUGUUGUCUAAAAGAAAAGAAGGCGCGUCCCUUUGGAGUGUUACAGUUUGAGG

.((((.((((((((.(((((((((((..(.((((((....((.....))..)))))).)..))))))))))).)))))))).)))). (MFE:-46.20)

>rheMac2_mml-mir-518d_chr19_59842537-59842623_+

UAUCGGGCUGUGACCCUCUAAAGGGAAGCGCUUUCUGUUGUGUGAAAGAAAACGAAGCGCUUCUCUUUGGAGCGUUACAGUUUGAGA

..((((((((((((.(((((((((((((((((((..(((...(....)..)))))))))))))))))))))).)))))))))))).. (MFE:-47.80)

>rheMac2_mml-mir-518a-2_chr19_59847852-59847938_+

UCUCGGGCUGUGACCCUACAAAGGGAAGCCCUUUCUGUUGUCUAAACGAAAAGAAAGUGCUUCUCUUUGCUGGGUUACGGUUUGAGA

((((((((((((((((..(((((((((((.((((((.((((....))))..)))))).)))))))))))..)))))))))))))))) (MFE:-50.40)

>rheMac2_mml-mir-517c_chr19_59849818-59849915_+

GAAGAUCUCCUCAUGCAGUGACACUCUAAAUGGCAGCACUGUCUGUGGUCUAAAACAAGAUCGUGCAGCCUUUUAGAGUGUUACUGUGUGAGAAAAGC

.........(((((((((((((((((((((.(((.((((.((((((........)).)))).)))).))).)))))))))))))))))))))...... (MFE:-49.80)

>rheMac2_mml-mir-519a-1_chr19_59861514-59861595_+

CUCAGGCUGUGACCCUCUACAGGGAGGCACUUUCUGUGGUCAGAAAAAGAAAGUGCACCCUUUUAGAGGAUUACUGUUUGAG

(((((((.((((.((((((.((((..(((((((((............)))))))))..)))).)))))).)))).))))))) (MFE:-40.50)

>rheMac2_mml-mir-527_chr19_59862788-59862874_+

UUUCAUGCUGUGACCCUACAAAGGGAAGCCCUUUCUGUUGUCUAAACUAAAAGAAAGUGCUUCUCUUUGCUGGGUUACGGUUUGAGA

(((((.((((((((((..(((((((((((.((((((...((....))....)))))).)))))))))))..)))))))))).))))) (MFE:-44.70)

>rheMac2_mml-mir-1283-2_chr19_59865973-59866059_+

CUCAUGCUGUGACUCUCUGGACAGAAGCGGUUUCUGUUGUCUGAAAGAAAACAAAUCGCUUCUGUCUAGAGUGUUUCGGUUUGAGAA

((((.((((.(((.((((((((((((((((((..((((.((.....)).)))))))))))))))))))))).))).)))).)))).. (MFE:-46.40)

>rheMac2_mml-mir-519a-2_chr19_59870526-59870609_+

UCUCAGGCUGUGACCAUCUACAGGGAAGCACUUUCUAUGGUCAGAAAAAGAAAGUGCAUCCUUUUAGAGUGUUACUAUUUGAGA

(((((((..(((((..((((.(((((.(((((((((.(........).))))))))).))))).))))..)))))..))))))) (MFE:-35.20)

>rheMac2_mml-mir-3177_chr20_1722639-1722720_+

CCGCAUGCCACGUGUACACAUAUGCCGGGCGCUGUCUCGAGACAUUCGCACAGCGCACUGCACUGGGGACACGUGGCACUGG

(((..((((((((((...(...(((.(((((((((..((((...)))).))))))).)))))...)..)))))))))).))) (MFE:-37.10)

>rheMac2_mml-mir-3677_chr20_2270926-2270985_+

UGCAGUGGCCAGAGCACUGCAGUGCUGGGCACAGGCUUCUCGUGGGCUCUGGCCAUGGCC

.((.(((((((((((.(..(((.((((....)).))..)).)..)))))))))))).)). (MFE:-32.40)

>rheMac2_mml-mir-3178_chr20_2543091-2543166_-

GCGGGGCGGGGCCGGAUCGGUCGAGAUCGGCCCUGCUGAUGACCGUCUCCCGUGCCCACGCCCCAAACGCUGUCUC

..(((((((((((((..(((((...((((((...)))))))))))....))).)))).))))))............ (MFE:-41.40)

>rheMac2_mml-mir-1972-1_chr20_15001540-15001616_+

UAUAGGCACGUGCCACCACACCUGGCUUAAAUGUGUCAUUUAAAAAUUCAGGCCAGGCACAGUGGCUCAUGCCUGUA

((((((((.(.(((((....((((((((.(((.............))).))))))))....))))).).)))))))) (MFE:-33.82)

>rheMac2_mml-mir-3179-1_chr20_16019315-16019375_+

CAGGAUCACAGACAUUUAAAUUUCACCCCUUCUACUAGAAAUUUAAAUGUCUGUGAUCCUG

((((((((((((((((((((((((.............)))))))))))))))))))))))) (MFE:-34.62)

>rheMac2_mml-mir-548w_chr20_24496088-24496161_+

GGUUGGUGCAAAAGUAAUUGCGGUUUUUGCCUUUAAACAUAAUGGCAAAACUCACAACUACUUUUGCCCCAAUC

((((((.(((((((((.(((.(..(((((((.(((....))).)))))))..).))).))))))))).)))))) (MFE:-31.40)

>rheMac2_mml-mir-3935_chr20_54557364-54557467_+

GGAUGUGUUCCUGUCCCAGAAGGAGCUGAUGGUUGUAUCUAUGAAGGUAAGCAUUUUUGUAGAUAGGAGCACCAGUCACCCUAAGCAAAGGCAGAGAAUGCUUA

....(((((((((((...(.(((.((((.((.((.((((((..(((((....)))))..)))))).)).)).))))...)))...)...))))).))))))... (MFE:-32.70)

>rheMac2_mml-mir-1538_chr20_67948161-67948218_-

GGGAACAGCAGCAACAAGGGCCUCGCUUCCUGCCGCCGCUGCCCGGGUUGCUGUUCCC

((((((((((((.....((((..(((........).))..))))..)))))))))))) (MFE:-30.00)

## 1.2 Mouse

>mm9_mmu-mir-1471_chr1_88534127-88534183_-

GCCCGAGUGUGGGGCCAGGUGUGGGGCUCGAGCACAGCUGGCUCCCAUUUGAGGGGC

(((((((((.((((((((.((((.........)))).)))))))))))))...)))) (MFE:-31.40)

>mm9_mmu-mir-1231_chr1_137351180-137351267_-

GUCAGUGUCUGGGCAGAGCUGCAGGAGAGAAGGGCAGGGCCGGACAUCUCUGCCACCCUGCCACCUGCCCUGUCUGUUCUGCCCACAG

.........((((((((((.(((((..((...(((((((..((.((....)))).)))))))..))..)))))..))))))))))... (MFE:-45.00)

>mm9_mmu-mir-3154_chr2_32173785-32173866_+

GGCCCCUCCUUCCCAACCCAGCUCCCGCUCACCCCUGCCACGUCAAAGGAGGCAGAAGGGGAGUCGGGAGCGGAGAGGGACC

((.(((((..(((.......((((((((((.((((((((.(......)..)))))..)))))).))))))))))))))).)) (MFE:-43.01)

>mm9_mmu-mir-1282_chr2_121276406-121276506_-

CCUUCUUCUCGUUUGCCUUUUUCUGCUUCUGCUGCAUGAUCUCCGAGUCCCUGGGGGUAGAGAUGAUGGGGCACUGGGAGGUACCAGAGGGCAAAAAGGAC

......(((..((((((((((..((((((((((.(((.(((((....(((....)))..))))).))).)))....)))))))..))))))))))..))). (MFE:-39.40)

>mm9_mmu-mir-2113_chr4_23553803-23553893_-

CUUUCAAAGCAAUGUUUGACAGGCACAGGGACAAAUCUUGUUAACAAGUAAGAGGAUUUGUGCUUGGCUCUGUCACAUGCCACUUUGAAAA

.((((((((..((((..(((((((.((((.(((((((((.(((.....))).))))))))).)))))).)))))))))....)))))))). (MFE:-36.70)

>mm9_mmu-mir-3115_chr4_136138068-136138132_-

UCUGAUGGGUUUACUAGUUGGUGGUAAAUUCUUGAGUCACCAGCUAUUAGGCCUUUAUGUCCAGA

(((((((((((((.((((((((((............)))))))))).)))))))....)).)))) (MFE:-21.50)

>mm9_mmu-mir-3666_chr6_15354981-15355091_+

ACUAAGGUCCGUCAGUUGUAAUGAGACCCAGUGCAAGUGUAGAUGCAGACUCGGUGGCAGAGUUCAGCAUUUCACACUGCCUGGUCUCUGUCACUCUAUUGAAUUAGAUUG

.....((((..(((((.((.((((((((.((.(((.((((((((((.(((((.......)))))..)))))).))))))))))))))).)).))...)))))....)))). (MFE:-36.20)

>mm9_mmu-mir-1179_chr7_86030400-86030490_+

GGCUAGGAAAGAAAAGCCAGUCUCUCAUUGGUUGGUAUGUGUCACUUCAUCAACCAAUAAGAGGAUGCCAUUUAUCCUUUCCUGACUAGCU

((((((((((((((.(.((..((((.((((((((((..(.....)...)))))))))).))))..)).).)))...)))))))....)))) (MFE:-27.40)

>mm9_mmu-mir-3174_chr7_87177387-87177482_+

GUUACCUAGGAGCUGGGUAGAAAUGCAGACUCCUGCCCCCCCCCCCCCCCCCGCAGAUUUGCUGAGGCUGCAUUUUAACCCCAGUACCAAGGAGGU

....(((.((.((((((..(((((((((.(((((((................)))).......))).)))))))))...)))))).)).))).... (MFE:-33.30)

>mm9_mmu-mir-3682_chr11_30795789-30795855_+

UAAUCUAUAUAUGUCUGUCUAUAACAGUAUACUCACAAUAUUGGUAUAGAUAGACAUAUAUAACUUU

.....((((((((((((((((((.((((((.......)))))).))))))))))))))))))..... (MFE:-27.00)

>mm9_mmu-mir-329-2_chr12_110951993-110952053_+

AGGAUGUGGAAGAGGGGUUUCUGUGUUUGAAACACAUUUGGUCAACCUCUCUUCCCAUCAG

..((((.(((((((((((((((((((.....)))))...))..)))))))))))))))).. (MFE:-27.30)

>mm9_mmu-mir-1271_chr13_54679499-54679585_+

CACCCAGGUGAGUGCUUGGCACCUGGUAAGCACUCAGUAAGUAUUUGAUGAGUGCCUACUGUGUGCCAAGACAUUGUGCUGAGGCUU

..(((((...((((((((((((.(((((.(((((((............))))))).))))).)))))))).))))...))).))... (MFE:-36.80)

>mm9_mmu-mir-3660_chr13_82374926-82375024_+

GAAGGAAGAACUAGACAAAAUUAAAAUCCUCCUCUGUCACUGUAAUAGUCCACUUCGGCCCGACAGCAGAGCAUUUUAACUUUGUCAAGUGUGUCUGCU

...(((...(((.((((((.(((((((.(((..(((((...((...((....))...))..)))))..))).))))))).)))))).)))...)))... (MFE:-27.90)

>mm9_mmu-mir-3613_chr14_62218173-62218267_-

CGAGUUUGGAUUGCUGUAUUUUUGUUGUUGUUGUUACUUGCAUUUUUCAAGAACAACAAAAACAAAAACACAGCCCAACCCUUCACACCACUUUG

.(((.((((...(((((.((((((((.((((((((.((((.......)))))))))))).)))))))).)))))))))...)))........... (MFE:-28.80)

>mm9_mmu-mir-3689a_chr15_13727869-13727946_-

ACUGAGUCGUCUGUUCUCCUAACUACUGAGUAGUCUGUUCUCCUAACUACUGAGUAGUCUGUUCUCCUAACUACUGAG

.((.(((.((..(.....(..((((((.((((((..(.....)..)))))).))))))..).....)..)).))).)) (MFE:-19.40)

>mm9_mmu-mir-2053_chr15_47784763-47784852_+

CUUGGCAUGUAAAUAUGGAUUUAAUUAACACUUACAACUUGUAAAGAGUGAAGCCUUAAGUGUUAAUUAAGCCUGUGUUUACAUAGCAAG

....((((((((((((((..((((((((((((((..((((.....)))).......))))))))))))))..)))))))))))).))... (MFE:-30.80)

>mm9_mmu-mir-3610_chr15_51823210-51823283_-

AAGAGCCGCGCCGUAACGGCCGCCAUCUUGUUUGUUUGAGUGAAUCGGAAAAGGAGGCGGCGGCCGUGGCGGUG

........((((((.(((((((((..(((.(((.(((((.....)))))))).)))..))))))))).)))))) (MFE:-38.70)

>mm9_mmu-mir-1234_chr15_76432652-76432750_-

GUAAGUGGGGUGCACUUUGGUCAAAGUGGGAUGUGCCUGGUGCCAGGCAGGAUGUGGUGAAGGCCAGGUAUUCAGUUUACAUGAUCAUGUUCCCCAUAG

....((((((.(((...((((((..(((((..(((((((((((((.(.....).))))....)))))))))....))))).))))))))).)))))).. (MFE:-37.50)

>mm9_mmu-mir-1281_chr15_81416563-81416616_+

AGGGGGCACCGGGAGGAGGUGAGUGUCUCUGGUCGCCUCCUCCUCUCCCCCUUU

((((((....((((((((((((.(......).))))))))))))..)))))).. (MFE:-34.20)

>mm9_mmu-mir-3618_chr16_18284643-18284730_-

UAAGCUGAGUGCAUUGUGAUUUCCAAUAAUUGAGGCAGUGGUUCUAAAAGCUGUCUACAUUAAUGAAAAGAGCAAUGUGGCCAGCUUG

(((((((..((((((((..(((.((.((((..(((((((..........)))))))..)))).)).)))..))))))))..))))))) (MFE:-29.20)

>mm9_mmu-mir-1248_chr16_23110826-23110931_+

UUCACCUUCUUGUAUAAGCACUGUGCUAAAAUUGCAGGAACUAAGAUUCUAUCUUGGUUUUUGUAAUAAUGCUAGCAGAGUACACACAAGAAGAAAAGUAACUGCA

.....((((((((....(.(((.(((((..(((((((((((((((((...))))))))))))))))).....))))).))).)..))))))))............. (MFE:-34.30)

>mm9_mmu-mir-1260b_chr9_random_423530-423608_-

CUCGGUUUAUCCCACCGUUAUCAUUACUGGUCAGCCGGUGCUGCUGAUGGUGAUGGUGAUAGUCUGGUGGAGGUGGUGG

......(((((((((((((((((((((((.(((((.......)))))))))))))))))).....))))).)))))... (MFE:-29.20)

# 2. pre-miRNAs predicted in SD pairs

## 2.1 Human

>hg19_hsa-mir-3675-2_chr1_16875409-16875482_+

GGAUGAUAAGUUAUGGGGGCUUCUGUAGAGAUUUCUAUGAGAACAUCUCUAAGGAACUCCCCCAAACUGAAUUC

((((....((((.((((((.((((.((((((((((.....))).))))))).))))..))))))))))..)))) (MFE:-27.90)

>hg19_hsa-mir-3675-2_chr1_17007750-17007823_+

GGAUGAUAAGUUAUGGGGGCUUCUGUAGAGAUUUCUAUGAGAACAUCUCUAAGGAACUCCCCCAAACUGAAUUC

((((....((((.((((((.((((.((((((((((.....))).))))))).))))..))))))))))..)))) (MFE:-27.90)

>hg19_hsa-mir-4252b_chr1_17044635-17044697_+

UGGGGGGCUGGCAGCUCAUCAGUCCAGGCCAUCUGGCCACUGGGUCGGCACCAGCGCCCAAUC

..(((.(((((..(((..(((((...((((....)))))))))...))).))))).))).... (MFE:-32.10)

>hg19_hsa-mir-3654-2_chrX_114938132-114938187_+

UUCAUGAGCUGCAAUCUCAUCACUGGAAUGUUCCAGUGACUGGACAAGCUGAGGAA

.((.(.((((....(((..((((((((....))))))))..)))..)))).).)). (MFE:-20.10)

>hg19_hsa-mir-3690-2_chrX_1412508-1412582_+

CCCACCUCCACCUGGACCCAGCGUAGACAAAGAGGUGUUUCUACUCCAUAUCUACCUGGACCCAGUGUAGAUGGG

((((.((.(((.(((..((((.(((((....(((.........)))....)))))))))..)))))).)).)))) (MFE:-28.20)

>hg19_hsa-mir-3690-2_chrY_1362508-1362582_+

CCCACCUCCACCUGGACCCAGCGUAGACAAAGAGGUGUUUCUACUCCAUAUCUACCUGGACCCAGUGUAGAUGGG

((((.((.(((.(((..((((.(((((....(((.........)))....)))))))))..)))))).)).)))) (MFE:-28.20)

>hg19_hsa-mir-3690-1_chrY_1362811-1362885_+

CCCAUCUCCACCUGGACCCAGCGUAGACAAAGAGGUGUUUCUACUCCAUAUCUACCUGGACCCAGUGUAGAUGGG

(((((((.(((.(((..((((.(((((....(((.........)))....)))))))))..)))))).))))))) (MFE:-32.80)

>hg19_hsa-mir-3198-2_chr12_54625181-54625260_-

GACUCUGCUCUCACUGUUCACCCAGCACUAGCAGUACCAGAUGGUUCUGUGGAGUCCUGGGGAAUGGAGAGAGCACAGUC

((((.(((((((.((((((.(((((.(((.((((.(((....))).))))..))).))))))))))).))))))).)))) (MFE:-44.50)

>hg19_hsa-mir-3648-2_chrUn_gl000220_107909-108088_+

CGCGACUGCGGCGGCGGUGGUGGGGGCAGCCGCGGGGAUCGCCGAGGGCCGGUCGGCCGCCCCGGGUGCCGCGCGGUGCCGCCGGCGGCGGUGAGGCCCCGCGCGUGUGUCCCGGCCGCGGUCGGCCGCGCUCGAGGGGUCCCCGUGGCGUCCCCUUCCCCGCCGGCCGCCUUUCUCGCG

(((((..((((((((((.((.((((((.((((((((((((.((..(((((((((((((((.(((((((((((((((.(((((((....))))..))).))))))).))).)))))..))))))))))).))))..))))))))))))))))))))..))))))).))))).....))))) (MFE:-141.60)

>hg19_hsa-mir-3687b_chrUn_gl000220_108280-108340_+

CGCGCGUGCGCCCGAGCGCGGCCCGGUGGUCCCUGCCGGACAGGCGUUCGUGCGACGUGUG

(((((((.(((.(((((((.(.((((..(...)..)))).)..))))))).)))))))))) (MFE:-36.50)

>hg19_hsa-mir-3648-2_chrUn_gl000220_151881-152060_+

CGCGACUGCGGCGGCGGUGGUGGGGGCAGCCGCGGGGAUCGCCGAGGGCCGGUCGGCCGCCCCGGGUGCCGCGCGGUGCCGCCGGCGGCGGUGAGGCCCCGCGCGUGUGUCCCGGCCGCGGUCGGCCGCGCUCGAGGGGUCCCCGUGGCGUCCCCUUCCCCGCCGGCCGCCUUUCUCGCG

(((((..((((((((((.((.((((((.((((((((((((.((..(((((((((((((((.(((((((((((((((.(((((((....))))..))).))))))).))).)))))..))))))))))).))))..))))))))))))))))))))..))))))).))))).....))))) (MFE:-141.60)

>hg19_hsa-mir-3687b_chrUn_gl000220_152252-152312_+

CGCGCGUGCGCCCGAGCGCGGCCCGGUGGUCCCUGCCGGACAGGCGUUCGUGCGACGUGUG

(((((((.(((.(((((((.(.((((..(...)..)))).)..))))))).)))))))))) (MFE:-36.50)

>hg19_hsa-mir-4273_chrUn_gl000222_15194-15277_-

UCCCCUGUGUGUGUUCUCUGAUGGACAGUAAGCCUUGACUUAUGGCUAAAUGCUUCUUCACAAUGGUCACAUGCAUAGGGCUUU

..((((((((((((((..((.((((.((((((((.........))))...))))..))))))..))..)))))))))))).... (MFE:-26.30)

## 2.2 Mouse

>mm8_mmu-mir-297a-7_chr7_10225343-10225418_-

AUAUGUAUGUAUGUAUGUAUGUGUGCAUGUGCAUGUGCAUGUAUGCAUAUUGUAUGUAUAUAUUAUGCAUACAUGU

(((((((((((((.(((((((..((((((((((((......)))))))).))))..)))))))))))))))))))) (MFE:-33.90)

>mm8_mmu-mir-344h_chr7_61618298-61618363_-

CAGUCAGGCUUCUGGCUAUAUUCCAGGACAUACCUGGUCCUGGGUAUAACCAAAGCCCGACUGUAU

(((((.(((((.(((.(((((.(((((((.......)))))))))))).)))))))).)))))... (MFE:-33.80)

# 3. Reference

1. Jiang P, Wu H, Wang W, Ma W, Sun X, et al. (2007) MiPred: classification of real and pseudo microRNA precursors using random forest prediction model with combined features. Nucleic Acids Res 35: W339-344.

2. Hofacker IL, Fontana W, Stadler PF, Bonhoeffer LS, Tacker M, et al. (1994) Fast folding and comparison of RNA secondary structures. Monatshefte für Chemie/Chemical Monthly 125: 167-188.
