# Supplementary figures and images for: MicroRNA Genes Derived from Repetitive Elements and Expanded by Segmental Duplication Events in Mammalian Genomes
Source: PLoS One. 2011 Mar 16;6(3):e17666. doi: 10.1371/journal.pone.0017666 (PMC3059204; doi:10.1371/journal.pone.0017666)

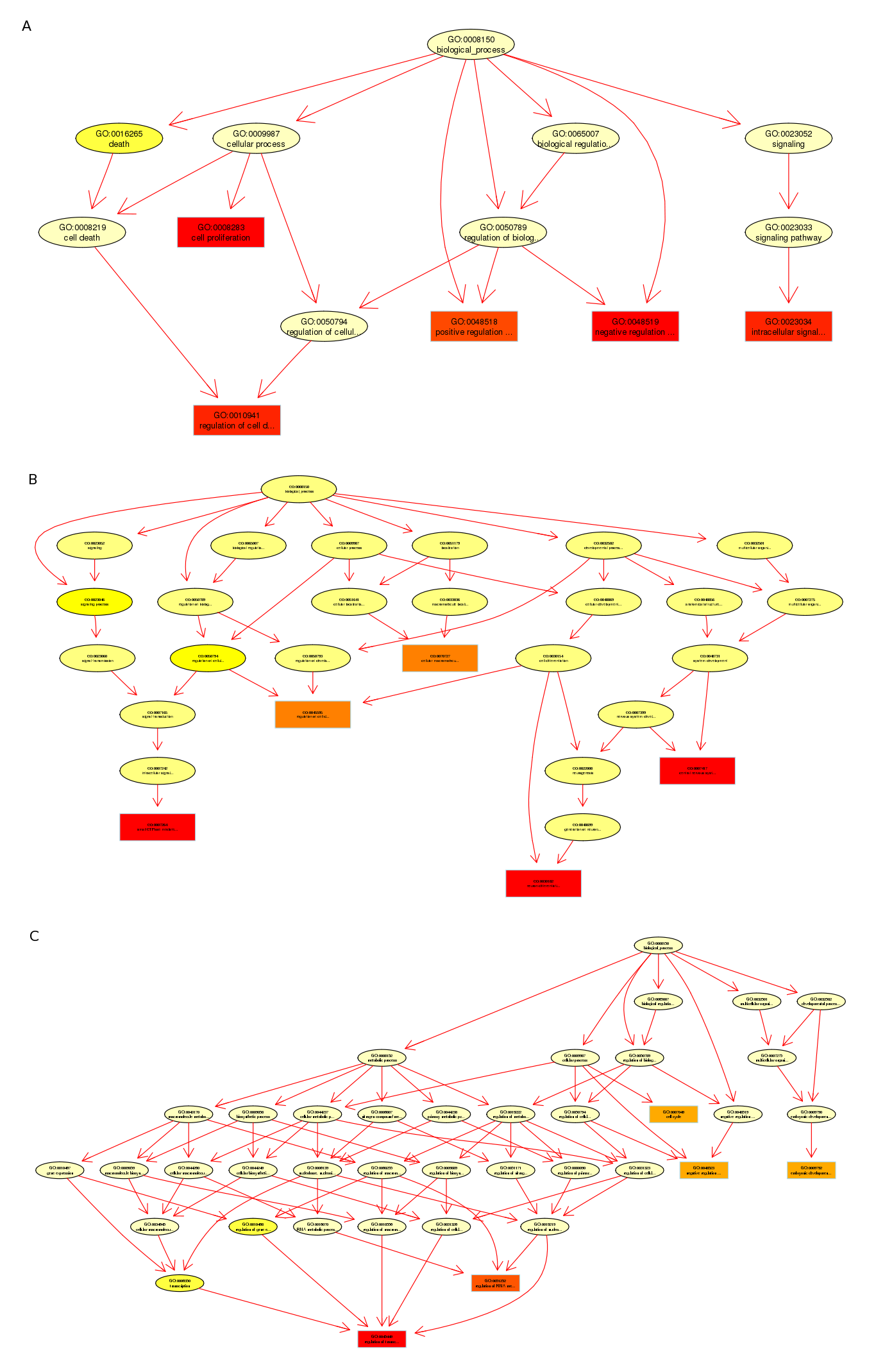

Supplement: Figure S1 — The topological GO graph for the enriched GO biology process terms of the target genes of common RrmiRs in the human, rhesus and mouse genomes. (A) human (B) rhesus (C) mouse. (TIF) [file pone.0017666.s002.tif]
